# A multifunctional nanocomposite hydrogel with controllable release behavior enhances bone regeneration

**DOI:** 10.1093/rb/rbad046

**Published:** 2023-04-28

**Authors:** Yingji Mao, Yiwen Zhang, Ying Wang, Tao Zhou, Bingxu Ma, Pinghui Zhou

**Affiliations:** Department of Orthopedics and Department of Plastic Surgery, The First Affiliated Hospital of Bengbu Medical College, Bengbu, Anhui 233004, China; Anhui Province Key Laboratory of Tissue Transplantation, School of Life Sciences, Bengbu Medical College, Bengbu, Anhui 233030, China; Department of Orthopedics and Department of Plastic Surgery, The First Affiliated Hospital of Bengbu Medical College, Bengbu, Anhui 233004, China; Department of Plastic Surgery and Burn Center, Second Affiliated Hospital, Plastic Surgery Institute of Shantou University Medical College, Shantou, Guangdong 515063, China; Department of Orthopedics and Department of Plastic Surgery, The First Affiliated Hospital of Bengbu Medical College, Bengbu, Anhui 233004, China; Department of Orthopedics and Department of Plastic Surgery, The First Affiliated Hospital of Bengbu Medical College, Bengbu, Anhui 233004, China; Department of Orthopedics and Department of Plastic Surgery, The First Affiliated Hospital of Bengbu Medical College, Bengbu, Anhui 233004, China; Department of Orthopedics and Department of Plastic Surgery, The First Affiliated Hospital of Bengbu Medical College, Bengbu, Anhui 233004, China; Anhui Province Key Laboratory of Tissue Transplantation, School of Life Sciences, Bengbu Medical College, Bengbu, Anhui 233030, China

**Keywords:** tissue engineering, nanocomposite multifunctional hydrogels, drug release, antibacterial capacity, bone regeneration

## Abstract

Autologous and allogeneic bone grafts remain the gold standard for repairing bone defects. However, donor shortages and postoperative infections contribute to unsatisfactory treatment outcomes. Tissue engineering technology that utilizes biologically active composites to accelerate the healing and reconstruction of segmental bone defects has led to new ideas for *in situ* bone repair. Multifunctional nanocomposite hydrogels were constructed by covalently binding silver (Ag^+^) core-embedded mesoporous silica nanoparticles (Ag@MSN) to bone morphogenetic protein-2 (BMP-2), which was encapsulated into silk fibroin methacryloyl (SilMA) and photo-crosslinked to form an Ag@MSN-BMP-2/SilMA hydrogel to preserve the biological activity of BMP-2 and slow its release. More importantly, multifunctional Ag^+^-containing nanocomposite hydrogels showed antibacterial properties. These hydrogels possessed synergistic osteogenic and antibacterial effects to promote bone defect repair. Ag@MSN-BMP-2/SilMA exhibited good biocompatibility *in vitro* and *in vivo* owing to its interconnected porosity and improved hydrophilicity. Furthermore, the multifunctional nanocomposite hydrogel showed controllable sustained-release activity that promoted bone regeneration in repairing rat skull defects by inducing osteogenic differentiation and neovascularization. Overall, Ag@MSN-BMP-2/SilMA hydrogels enrich bone regeneration strategies and show great potential for bone regeneration.

## Graphical Abstract

**Figure:**
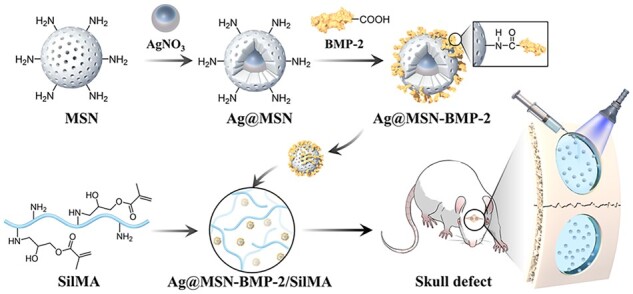

## Introduction

Bone is a mineralized connective tissue with a unique self-healing capacity. However, the health of people is significantly threatened by bone defects brought on by trauma, tumors, inflammation, osteonecrosis and osteoporosis [1]. An increase in defect size dramatically increases the probability of bacterial infection, and the improper use of antibiotics (especially overuse) has led to the emergence of many multidrug-resistant bacteria that further hinder the self-repair of bone defects [2]. Thus, achieving successful *in situ* bone regeneration in clinical practice is a significant challenge. Currently, autologous, and allogeneic bone grafts remain the gold standard for treating bone defects; however, their clinical applications are usually restricted by donor shortage, donor site pain, infection and bleeding [3, 4]. Researchers are therefore more motivated and interested in creating artificial bone substitutes that can replace these conventional bone regeneration techniques. Recent efforts involve developing new bioactive materials with great biocompatibility, biodegradability, antibacterial capacity and osteogenic properties [5, 6].

*In situ* bone tissue engineering is regarded as a viable alternative to overcome the disadvantages of conventional bone grafting [7]. It aims to create an appropriate microenvironment that mimics natural tissue structure for regeneration [8]. Porous three-dimensional (3D) scaffolds can form an appropriate microenvironment for cells to grow and create the extracellular matrix (ECM) to initiate tissue regeneration [9]. Hydrogels are ideal scaffolds that mimic the natural ECM, both structurally and compositionally [10, 11]. Hydrogels have a 3D polymer network with high water absorption that provides cells with a 3D microenvironment to imitate the native ECM and facilitate nutrient transport [12]. Specifically, injectable hydrogels characterized by their capacity to fill irregular sites have become ideal biomaterials for inducing *in situ* bone regeneration [13]. Various hydrogels, including hyaluronic acid (HA) [14], collagen [15] and synthetic polymers [16] are extensively studied in tissue engineering. Meanwhile, silk fibroin (SF) is a natural protein composed of 18 amino acids that can lead to a lower inflammatory response and facilitate cellular adhesion and proliferation compared to other biomaterials like polylactic acid or collagen. It is considered a standard biomaterial for bone regeneration because of its biocompatibility, biodegradability and mechanical properties [17, 18]. In particular, silk fibroin methacryloyl (SilMA) forms a 3D structured hydrogel by photo-crosslinking that is biocompatible, shows excellent mechanical properties and has a controlled biodegradation rate [19–21]. This hydrogel has attracted worldwide attention for biotechnological and biomedical applications. The SilMA hydrogel is a scaffold possessing mechanical properties for bone regeneration; however, it fails to mimic natural bone tissue and lacks osteogenic factors and other mineral ions that promote bone mineralization [21]. Therefore, *in situ* bone regeneration from pure SilMA hydrogels is difficult.

Bionic inorganic particles, such as silicates, hydroxyapatite (HAp), manganese-doped bioresorbable ceramic scaffolds and titanium compounds are commonly used during hydrogel preparation for better organic/inorganic compatibility to reproduce the chemistry of genuine bone [22–25]. Various growth factors, such as bone morphogenetic proteins (BMPs), transforming growth factor β (TGF-β) and insulin-like growth factor (IGF) were added to improve the osteoconductive and osteoinductive activities of composite hydrogels [26]. BMPs are a special type of bone growth factor that induces bone and cartilage formation. They are also the most often employed cytokines that encourage bone marrow mesenchymal stem cells (BMSCs) to differentiate into osteoblasts [27]. A large number of BMPs’ short peptide sequences (including BMP-2, BMP-7 and BMP-9) have a similar biological activity to that of full-length BMPs. BMP-2 is a well-known osteoinductive growth factor that stimulates the differentiation of BMSCs into osteoblastic cell lines for bone regeneration [28]. However, it is susceptible to rapid *in vivo* degradation by proteases, and high doses of this growth factor may have negative effects, including the development of ectopic bones, immunological reactions and even cancer [7]. Therefore, it is essential to immobilize BMP-2 in nanoparticles (NPs) to maintain its biological activity and control its release.

Mesoporous silica nanoparticles (MSNs), which have a high surface area, stability, targeting and biocompatibility, have generated a lot of attention in the biomedical field [29, 30]. These particles were proposed by Nooney et al. at the start of the 21st century and were characterized according to their: (1) tiny size with a high bulk surface area; (2) uniform distribution of pore channels with adjustable pore size; (3) big pore volume and (4) a large number of silica hydroxyl groups dispersed across the surface that may be modified to drug delivery systems with the sustained release [31, 32]. *In vitro*, studies have demonstrated the efficient capacity of MSN to deliver biomolecules such as antibiotics and proteins into cells, with mesoporous properties such as pore size, volume and surface area determining the loading and delivery efficiency [33]. BMP-2 loaded onto MSN has a greater osteoinductive effect on human mesenchymal stem cells compared with BMP-2 alone [34]. However, artificial bone grafts with a single function are insufficient to repair infected bone defects. Some studies have used antimicrobial components such as Ag NPs and antibiotics as surface coatings to enhance the antimicrobial capacity of materials. For example, hydrogels with silica and Ag NPs were fabricated to induce osteogenic differentiation of infected bone defects [35].

The highly reactive surface of Ag provides many adsorption and reaction sites with proteins, nucleic acids and other molecules to enable close contact with viruses. Small particle-sized nanosilver has a larger surface area and provides higher antibacterial activity [36]. Additionally, nanosilver is an effective antibacterial agent against bacterial drug resistance [37, 38]. However, its instability makes it difficult to preserve activity during application. Therefore, loading unstable nanosilver into inorganic carriers is a feasible and ideal approach to constructing antimicrobial agents.

**Figure 1.**
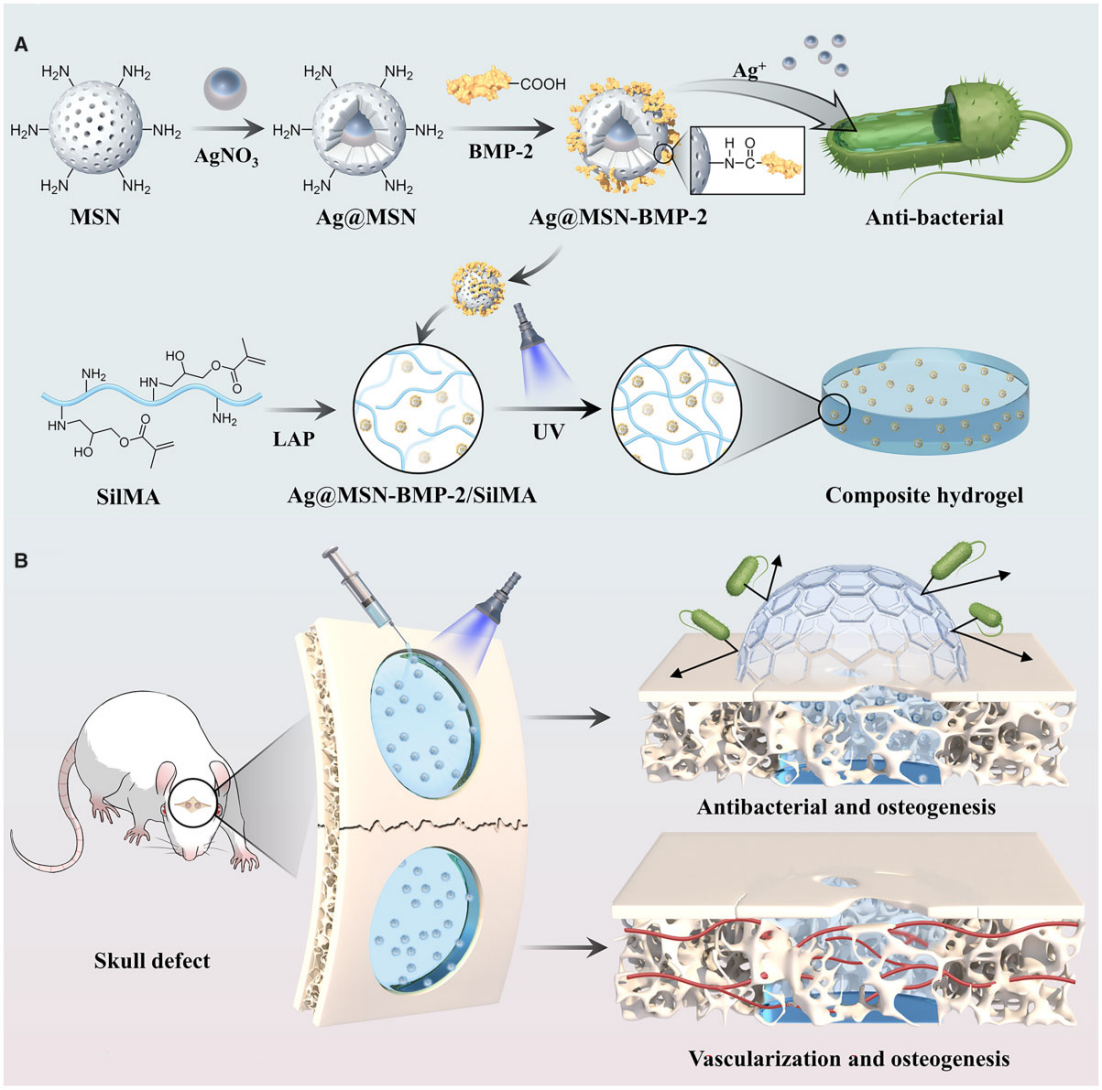
Schematic diagram of Ag@MSN-BMP-2/SilMA hydrogel to promote healing of tissue defects. (**A**) Preparation of Ag@MSN-BMP-2/SilMA. (**B**) Ag@MSN-BMP-2/SilMA hydrogel was injected into a rat skull defect model to exert antibacterial and osteogenic functions.

In this study, a novel multifunctional nanocomposite hydrogel was prepared and developed for *in situ* bone regeneration (Fig. 1). Ag@MSN-BMP-2 was synthesized by combining Ag-loaded MSN (Ag@MSN) with BMP-2 polypeptide, followed by covalent binding and introduction of Ag@MSN-BMP-2 into SilMA, then photo-crosslinking to obtain an Ag@MSN-BMP-2/SilMA nanocomposite hydrogel. This study explored the ability of Ag@MSN-BMP-2/SilMA to induce differentiation of BMSCs into osteoblasts, investigated the synergistic antibacterial effect of Ag@MSN *in vitro* and *in vivo*, and evaluated the *in vivo* biodegradability of the composite hydrogel and its osteogenic ability *in situ* in a rat skull defect model. This novel bioactive nanocomposite hydrogel with osteogenic antibacterial properties may present a viable technique for *in situ* bone regeneration.

## Materials and methods

### Synthesis and characterization of MSN and Ag@MSN

All the materials and their sources can be seen in the Supplementary Data. The preparation of MSN and Ag@MSN is shown in the Supplementary Data. Particle characterization refers to the Supplementary Data for details.

### Preparation of multifunctional nanocomposite hydrogels

#### Synthesis of Ag@MSN/BMP-2

BMP-2 peptide was covalently grafted onto the surface of Ag@MSN using a crosslinking reagent to prepare BMP-2 peptide-functionalized Ag@MSN/BMP-2. Initially, in a sterile phosphate-buffered saline (PBS) solution (pH 7.4), 200 g of BMP-2 peptide was added, followed by 0.2 mmol of EDC and 0.5 mmol of NHS. At room temperature, the mixture was stirred for 30 min, followed by the addition of 20 ml PBS containing 20 mg sterilized Ag@MSN and stirring for 24 h. To get rid of extra peptides, EDC and NHS, the solution was centrifuged and thoroughly washed with deionized water. The prepared Ag@MSN/BMP-2 was stored at 4°C [39]. The concentration of the BMP-2 solution was determined before and after loading and the following equation was used to calculate the drug-loading capacity:


$$\mathrm{BMP}-2\;\mathrm{loading}\;\mathrm{capacity}\;=\frac{\mathrm{weight}\;\mathrm{of}\;\mathrm{BMP}-2\;\mathrm{in}\;\mathrm{carriers}}{\mathrm{weight}\;\mathrm{of}\;\mathrm{Ag}@\mathrm{MSN}/\mathrm{BMP}-2\;\mathrm{in}\;\mathrm{carriers}}\times 100\%.$$

The preparation of BMP-2-FITC and the effect of the cell morphology of Ag@MSN-BMP-2 are shown in the Supplementary Data.

#### Preparation of SilMA hydrogel

The preparation of SilMA hydrogel is shown in the Supplementary Data.

The preparation of the photo-crosslinking SilMA hydrogel: 0.5 g lithium phenyl-2,4,6-trimethylbenzoyl phosphinate (LAP, Suzhou Intelligent Manufacturing Research Institute) was dissolved in 20 ml PBS in a water bath at 40–50°C for 15 min with periodic vigorous shaking during this period. Two grams of SilMA was then added and dissolved at 25°C for 1 h, protected from light, to prepare 10% SilMA, which was then sterilized with a 0.22 μm sterile needle filter.

### Material characterization

#### Nuclear magnetic resonance

Silk fibroin and SilMA were analyzed using ^1^H nuclear magnetic resonance (NMR) with a Bruker 400 MHz instrument to assess the binding of methacrylic anhydride (MA) to SF. The MestReNova software was used to analyze the data. The degree of substitution (DS) of SilMA was by dividing the integrated area of the methylene peaks at 5.52 and 5.90 ppm by that of the peaks at 2.75–2.85 ppm according to the equation below [40].


$$\;\mathrm{DS}\;=\frac{A(5.52\&5.9)/2}{A(2.75-2.85)/5}\times 100\%.$$

#### Scanning electronic microscopy and energy dispersive spectroscopy

Composite hydrogels with a diameter of 1 cm and a thickness of 5 mm were prepared and frozen at −20°C for 3–5 h and −80°C for 1–2 h. The frozen samples were freeze-dried for approximately 18 h. The freeze-dried samples were cut into thin and uniform slices and analyzed by scanning electronic microscopy (SEM) (Zeiss Gemini 300, Germany) to observe the morphology of the composite hydrogel. The elemental distribution was analyzed using energy dispersive spectroscopy (EDS).

#### Young’s modulus and water contact angle

Mechanical strength and hydrophilicity of nanocomposite hydrogels were performed using Young’s modulus and water contact angle (WCA). Refer to the Supplementary Data for details.

#### Swelling rate

The nanocomposite hydrogels were lyophilized, soaked in ultrapure water and placed in a shaker incubator (100 rpm) at 37°C. They were weighed at fixed time points to evaluate their swelling rate.

#### Release of BMP-2 peptide in vitro

Ag@MSN/BMP-2/SilMA was placed in PBS solution (5 ml, pH 7.4) in a shaker incubator (100 rpm) at 37°C to investigate the *in vitro* release of the BMP-2 peptide. The samples were centrifuged at 12 000 rpm at fixed time points, and 2 ml of the supernatant was collected to measure the BMP-2 peptide release profile using ultraviolet–visible (UV–vis) spectroscopy at 495 nm.

#### Release of Ag^+^ in vitro

Ag@MSNs were suspended in a Luria-Bertani medium to obtain a stable dispersion and incubated at 37°C. The adsorption value was plotted at 417 nm using UV–vis spectroscopy at fixed time points to plot the release profile of Ag^+^.

### *In vitro* experiment

#### Cell culture

The methods of extraction, culture and identification of BMSCs were previously reported by our research group, and the third passage of BMSCs was used for this study [39, 41]. The cells were placed in DMEM/F12 medium containing 10% serum and 1% penicillin–streptomycin solution and cultured in an incubator at 37°C with 5% CO_2_. The medium was changed every 2 days. The prepared composite hydrogels were injected into a cell culture dish. The mixed solution was exposed to 365 nm UV light for 30 s and immersed in a cell culture medium.

#### Cell proliferation

To determine the optimal concentration of nanomaterials, different concentrations (50, 100, 200 and 400 ng/ml) of MSN, Ag@MSN and Ag@MSN-BMP-2 were selected to co-incubate with BMSCs at a density of 5 × 10^4^/ml, respectively. On the third day, 10% of the total volume of cell counting kit-8 (CCK-8) was added and incubated for 2 h at 37°C in the dark, the supernatant was transferred and detected at an optical density (OD) of 450 nm.

#### Live/dead staining

The hydrogels were divided into four groups: SilMA, MSN/SilMA, Ag@MSN/SilMA and Ag@MSN-BMP-2/SilMA to observe the effect of the composite hydrogels on cell activity. The density of 5× 10^4^/ml BMSCs encapsulated in the hydrogels were cultured *in vitro* for 1, 4 and 7 days in each hydrogel. The samples were washed with PBS, then incubated for 40 min at 37°C and 5% CO_2_ using a calcein AM/PI double staining kit. The samples were rinsed thrice with PBS and photographed using a confocal laser scanning microscope (CLSM, Olympus Corporation, Japan).

#### Osteogenesis differentiation

The third passage of BMSCs was cultured in six-well plates at a density of 5 × 10^4^/ml to evaluate the osteogenic induction ability of NPs on BMSCs. The osteogenesis-induction medium (OM; Cyagen, China) was refreshed every 3 days when the cell density was 80%. The cells were co-cultured with 100 ng/ml MSN, Ag@MSN or Ag@MSN-BMP-2 for 7 days. Cells were cleaned with PBS, fixed with paraformaldehyde, and then stained with alkaline phosphatase (ALP; Yeasen, China). The ECM mineralized with calcium nodules was visualized 14 days after osteogenic induction using Alizarin Red S (ARS) staining (Cyagen, China).

The hydrogels were stained with ALP and ARS to evaluate the osteogenic differentiation ability of the multifunctional nanocomposite hydrogel scaffolds loaded with BMSCs.

#### Bacterial inhibition ring test

*Escherichia coli* (*E.coli*) and *Staphylococcus aureus* (*S.aureus*) were used as models for the bacterial inhibition ring test to assess the antibacterial activity of the multifunctional nanocomposite hydrogels. The bacterial solution was adjusted to 10^6^ CFU/ml and was uniformly distributed on the surface of the agar medium for inoculation. The sterilized samples were then applied to the surface of the agar medium with sterile forceps and gently pressed to make full contact between the samples and the medium. The dishes were incubated at 37°C for 12 h. The samples were removed and photographed. The inhibition range was measured using digital Vernier calipers, and inhibition curves were plotted.

### *In vivo* experiments

#### Experimental animals

Rats were acquired from Shushan Experimental Animal Center (Hefei, China). All animal experiments were granted permission by the Ethics Committee of the Medical Faculty of Bengbu Medical College (approval number: 2021272). Six-week-old male SD rats (200–250 g) and C57BL/6 mice aged 4 weeks (30 g) were used. All animals were housed in a suitable environment for 7 days before subsequent experiments.

#### In vivo antibacterial activity

Sprague Dawley rats (*n* = 50) were randomly divided into five groups: control, SilMA, MSN/SilMA, Ag@MSN/SilMA and Ag@MSN-BMP-2/SilMA (10 rats per group). The rats were anesthetized, shaved, disinfected, and a 10-mm circular skin incision was made. The incisions of rats in different groups were injected with different hydrogels and photocrosslinked for 30 s, except for the control group. All rats were reared in separate cages without antibiotic injection. The tissues around the incisions were selected for immunofluorescence analysis on days 4, 7 and 14.

Skin sections were incubated overnight at 4°C with an anti-*S.aureus* primary antibody (anti-*S.aureus* antibody, ab68950, Abcam). The samples were then incubated with a fluorescently coupled secondary antibody (Molecular Probes, Life Technologies, USA) for 1 h at room temperature. Finally, the nuclei were re-stained with 4′,6-diamidino-2-phenylindole (DAPI, 1:200). After blocking, the sections were analyzed under CLSM. Three images were selected and immunostained sections were analyzed with Image-J software. Five regions were calculated for each image, analyzed and plotted using Graph Pad Prism. Each sample was repeated three times.

#### In vivo degradability and biocompatibility

The fluorescently labeled dye with an excitation wavelength of 550 nm (EFL-DYE-UF-ENE-R) was dissolved in LAP at 5 mg/ml to prepare fluorescent hydrogels. All samples were divided into SilMA, MSN/SilMA, Ag@MSN/SilMA and Ag@MSN-BMP-2/SilMA groups. The hydrogels were made into cylinders with a diameter of 1 cm and a height of 5 mm using a mold. Mice were anesthetized, then the backs of the mice were shaved and sterilized. The samples were implanted into the backs of the mice (*n* = 40, 10 mice per group). Photographs were taken immediately after the operation (day 0) and at weeks 1, 4 and 8 using a subcutaneously small-animal live imaging system (Bruker) to observe and record hydrogel degradation in different groups.

All samples in SilMA, MSN/SilMA, Ag@MSN/SilMA and Ag@MSN-BMP-2/SilMA groups were crosslinked to assess the biocompatibility of the composite hydrogels by UV light *in vivo*, and subcutaneously implanted into the back of rats (*n* = 40, 10 rats per group). The rats were anesthetized at 1, 4 and 8 weeks after implantation, and each implanted hydrogel scaffold was removed and weighed to plot the degradation curve. Skin tissue fixed, embedded in paraffin, and sectioned. Hematoxylin–eosin (H&E) staining and CD68 (DF7518, Affinity) immunofluorescence were subsequently performed to assess the *in vivo* biocompatibility of the hydrogels.

#### Establishment of a skull defect model in rats

Sprague Dawley rats (*n* = 100) were randomly divided into five groups (20 rats per group): control, SilMA, MSN/SilMA, Ag@MSN/SilMA and Ag@MSN-BMP-2/SilMA. A skull defect model was established as previously reported [5, 7]. Rats were anesthetized and sterilized with iodophor. The skin was cut along the midline of the skull layer by layer with a surgical blade under aseptic conditions and on both sides of the sagittal suture of the skull. Two symmetrical bone defect models with a diameter of 5 mm were established with a circular drill bit. The hydrogels were then added to the defects and crosslinked *in situ* using UV light, followed by suturing the skin of the rat’s head and wiping the blood from the rat’s skin surface with iodophor. Each rat was continuously postoperatively injected with intramuscular penicillin (20 × 4^10^ U) for 3 days to prevent infection. The rats were postoperatively euthanized at weeks 4 and 8 and cranial bone samples were used for experiments.

#### Micro-computed-tomography analysis

Five rat cranial bone samples were surgically removed from each group at weeks 4 and 8 and preserved in 4% formalin to assess bone formation using micro-computed tomography (micro-CT). The specimens were sequentially scanned using a dual-energy X-ray bone densitometer according to the group. The bone mineral density (BMD) and the ratio of new bone formation to total volume (BV/TV) were analyzed.

#### Revascularization capacity

Angiographic analysis was performed 4 weeks after surgery. Five rats were selected from each group, and the left ventricle was exposed and perfused with sodium heparin solution followed by paraformaldehyde containing 2% barium sulfate using a vascular catheter under general anesthesia. Angiography was performed after skull removal to assess the angiogenic ability of the composite hydrogel.

#### Histological analysis

The collected cranial tissues were initially immersed in formalin for 24–48 h. Subsequently, the tissues were placed in ethylenediaminetetraacetic acid (EDTA) decalcifying solution for at least 30 days, and the EDTA decalcifying solution was changed at least once daily. Paraffin-embedded sections of decalcified skulls were stained with H&E and Masson’s trichrome stain, and the bone tissue slices were processed by immunohistochemistry staining. The color development of OCN (DF12303, Affinity), RUNX2 (AF5186, Affinity) and CD31 (AF61911, Affinity) was obtained using 3,3′-diaminobenzidine tetrahydrochloride (DAB; Solarbio, China), and the sealed sections were observed under a fluorescent microscope (Observer Z1) and photographed for documentation. Lastly, bone formation ability was evaluated by intraperitoneally injecting rats with Alizarin Red (35 mg/kg) and Calcein (30 mg/kg) at 2, 4 and 6 weeks. The skulls were collected after 8 weeks of sequential fluorescent labeling. Paraffin-embedded sections were prepared and 50 μm-thick uncalcified sections were observed by CLSM for imaging and counting.

Finally, the images were captured using a fluorescence microscope (Observer Z1). Three images were selected and the fluorescence intensity of the immunofluorescence-stained sections was analyzed using Image-J software. Counting, analysis and plotting were performed for each image using GraphPad Prism software.

### Statistical analysis

Data are expressed as mean ± standard deviation. All data were processed using GraphPad Prism (GraphPad Software, Inc., USA), and differences were assumed to be statistically significant at a *P*-values <0.05.

## Results

### Characterization of Ag@MSN-BMP-2/SilMA

**Figure 2.**
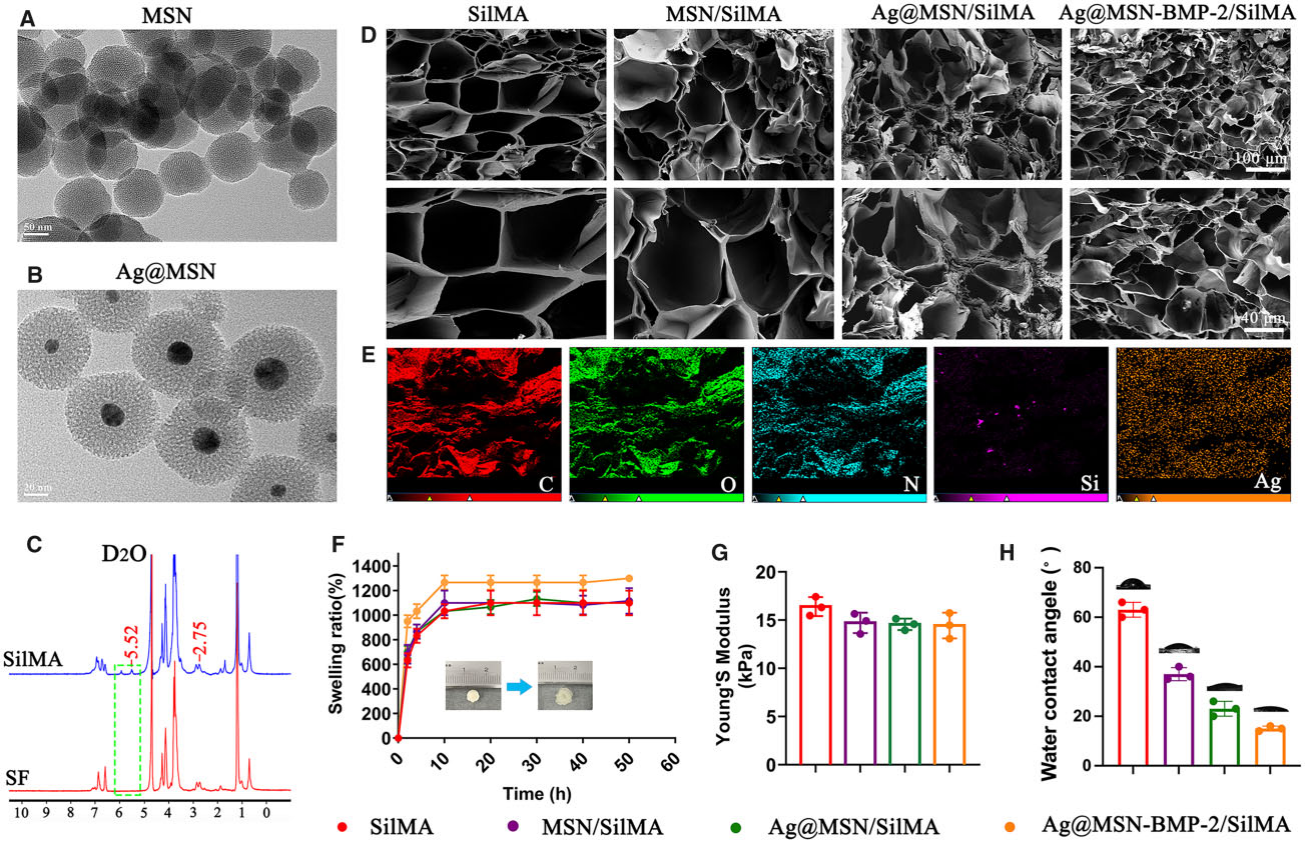
Morphology and characterization of nanocomposite hydrogel scaffolds. (**A** and **B**) TEM images of MSN and Ag@MSN. (**C**) ^1^H NMR spectra of SF and SilMA. (**D**) SEM images of the inner porous structures of SilMA, MSN/SilMA, Ag@MSN/SilMA and Ag@MSN-BMP-2/SilMA. (**E**) EDS images of C, O, N, Si and Ag of Ag@MSN-BMP-2/SilMA. (**F**) Equilibrium swelling property of freeze-dried composite hydrogels. (**G**) Young’s modulus and (**H**) WCA of nanocomposite hydrogels.

The MSN had a spherical morphology with an average diameter of 120 nm from TEM images (Fig. 2A). The TEM image of Ag@MSN (Fig. 2B) showed that the AgNPs had spherical core-shell structures with a mean diameter of 85 nm and a nucleus with a mean diameter of 15 nm embedded in the center of the Ag@MSN NPs (Supplementary Fig. S1). The typical Fourier transform infrared spectroscopy (FTIR) signals at ∼1487 and ∼1641 cm^−1^ corresponded to the absorption of amide I and II bands, respectively, of the peptide chain on the surface of the MSN [42]. The FTIR plot of Ag@MSN showed typical signals of Si–O–Si groups in the antisymmetric stretching vibrational band (1074 cm^−1^), a symmetrical stretching vibrational band (800 cm^−1^) and a vibrational bending band (457 cm^−1^); this indicated that Ag@MSN was successfully prepared (Supplementary Fig. S2) [43]. Meanwhile, the fluorescence microscopy images of Ag@MSN-BMP-2-FITC demonstrated the successful grafting of BMP-2 (labeled KIPKASSVPTELSAISTLYL) onto Ag@MSNs (Supplementary Fig. S3). The CCK-8 results showed that 100 ng/ml NPs of each group did not affect the activity of BMSCs compared with the control group (Supplementary Fig. S4). Therefore, this NP concentration was selected for subsequent experiments. Co-culture of Ag@MSN-BMP-2-FITC with BMSCs for 48 and 72 h showed uniformly distributed BMSCs with a clear cytoskeleton according to confocal microscopy images. This indicated that the addition of NPs did not affect the morphology of BMSCs (Supplementary Fig. S5).

SilMA was prepared by a substitution reaction between the amino group of SF and methyl methacrylate. The functional groups of SilMA were cross-linked with LAP by irradiation with UV light. The grafting of MA to SF was demonstrated by ^1^H NMR spectroscopy after dissolving the synthesized monomer hydrogels in D_2_O. The new vinyl proton peaks in SilMA at 5.52 ppm are supported by the established values (Fig. 2C). The degree of chitosan methacrylation was 24.36% by integrating the peak areas in the ^1^H NMR spectra. The structure of the different hydrogels containing NPs changed after freeze-drying according to the SEM images (Fig. 2D). The pore size of the hydrogels containing NPs was significantly reduced after freeze-drying compared with that of the SilMA group. In addition, the elements C, O and N representing the SF, Si (silica) and Ag (representing Ag nanoparticles) were uniformly dispersed inside the hydrogel. This proved that the SilMA hydrogel was capable of *in situ* loading of uniformly dispersed NPs (Fig. 2E).

The release profiles of Ag and BMP-2 from Ag@MSN-BMP-2/SilMA were explored (Supplementary Figs S6 and S7). The *in vitro* release profiles of Ag were obtained from the UV–Vis absorbance measurements of Ag changes. Ag was rapidly released in the first 100 h, followed by a relatively slow release in the later period. The loading rate of BMP-2 was 14.9% based on the loading rate equation. The BMP-2 peptide was slowly released after SilMA wrapping and continued to be slowly released for 28 days. This suggests that covalent bonding can slow the release of peptides.

The addition of Ag@MSN-BMP-2 to SilMA significantly affected the swelling rate (Fig. 2F). The swelling ratio of Ag@MSN-BMP-2/SilMA reached over 1200 wt% after 10 h, whereas the other groups peaked at over 1000 wt% after 10 h. The mechanical strength of the SilMA and composite hydrogels was evaluated by determining Young’s modulus; unmodified SilMA had the highest mechanical strength: 16.83 ± 0.56 kPa (Fig. 2G). Young’s modulus of MSN/SilMA, Ag@MSN/SilMA and Ag@MSN-BMP-2/SilMA was 14.88 ± 0.98, 14.62 ± 0.52 and 14.10 ± 1.12 kPa, respectively. This indicated that the mechanical strength of the composite hydrogels changed after NP addition. However, the changes among the groups of composite scaffolds with NPs were negligible. Additionally, the WCA of SilMA, MSN/SilMA, Ag@MSN/SilMA and Ag@MSN-BMP-2/SilMA was 62.3° ± 0.55°, 38.4° ± 0.65°, 21.4° ± 0.65° and 17.9° ± 0.55° (Fig. 2H). These results indicated that the hydrophilicity of the hydrogels improved after the addition of NPs, and the composite hydrogels had a high absorption capacity. As a result, when the water droplets made contact with the scaffold surface, they were instantly absorbed.

### Cytocompatibility of multifunctional nanocomposite hydrogels *in vitro*

**Figure 3.**
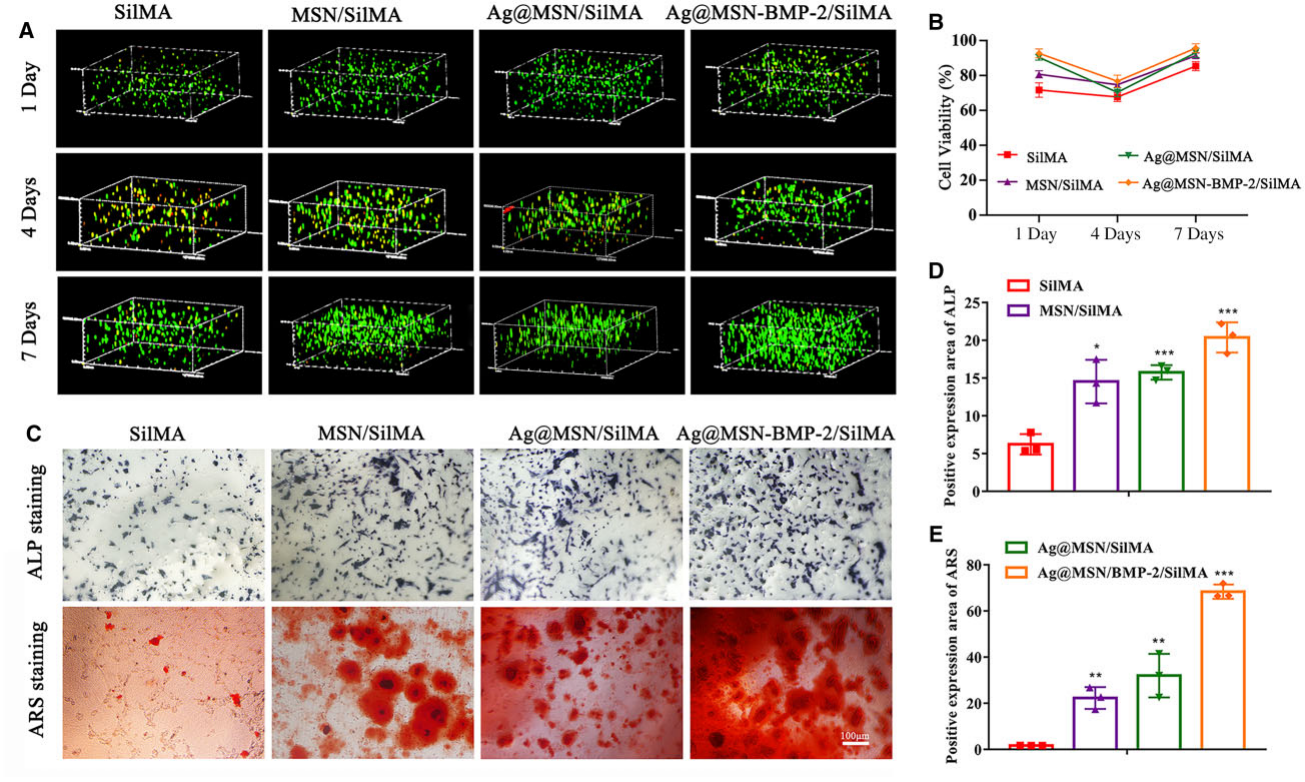
Evaluation of nanocomposite multifunctional hydrogels *in vitro*. (**A**) The live/dead staining of BMSC cultures on SilMA, MSN/SilMA, Ag@MSN/SilMA and Ag@MSN-BMP-2/SilMA after 1, 4 and 7 days of culture. (**B**) Cell viability of BMSC cultures on different multifunctional nanocomposite hydrogels after 1, 4 and 7 days of culture. (**C**) The immunodetection of ALP and ARS in BMSCs cultured on the hydrogel surface after induction for 7 and 14 days. (**D** and **E**) Quantitative analysis of ALP and ARS expression (*P <* *0.05, **P <* *0.01 and ***P <* *0.001 compared with the SilMA group).

Live/dead staining was performed at 1, 4 and 7 days to determine cell survival in the different hydrogels. Most BMSCs survived (green fluorescence) and only a few died (red fluorescence) on day 1 (Fig. 3A). This indicated that all hydrogels had considerable biocompatibility. The proportion of dead cells gradually increased in the different hydrogels on day 4. The cells in each group of hydrogels maintained viability at ∼80–90% on day 7 (Fig. 3B). In conclusion, nanocomposite hydrogels provided a favorable environment for the survival of BMSCs.

### Osteogenesis promoted by multifunctional nanocomposite hydrogels *in vitro*

Alkaline phosphatase is an early osteogenic marker that is abundant in the early stages of BMSC osteogenic differentiation. Cells were stained for ALP on day 7 after induction. Cells in the Ag@MSN-BMP-2 group had more blue clumpy precipitates with a deeper color compared with the other groups. Calcium nodules are late osteogenesis markers and they were stained with Alizarin red dye on the day after induction. The number of orange nodules and the degree of staining gradually increased in the Ag@MSN-BMP-2 group; this correlated with the ALP staining results. The combination of BMP-2 and Ag@MSN resulted in significantly higher calcium deposition in the Ag@MSN-BMP-2 group than in the control, MSN and Ag@MSN groups (Supplementary Fig. S8).

The potential osteoinductivity of nanocomposite multifunctional hydrogels was investigated by determining the osteogenic differentiation of BMSCs on various hydrogel scaffolds at different durations. This was performed by specific biochemical staining and quantitation to detect early-stage and late-stage osteogenic markers: ALP and calcium nodules, respectively. ALP staining showed clusters of blue–violet nodular deposits around the BMSCs after 7 days of induction in the Ag@MSN-BMP-2/SilMA group and Alizarin Red staining was positive for calcium staining on day 14. There were a few orange–red mineralized nodules in the SilMA, MSN/SilMA and Ag@MSN/SilMA groups, whereas the Ag@MSN-BMP-2/SilMA group showed a higher number of mineralized nodules with a deeper color and partial fusion (Fig. 3C). Statistical analysis suggested the same trend (Fig. 3D and E). Comparative analyses indicated that Ag@MSN-BMP-2/SilMA promoted osteogenic differentiation of BMSCs to a greater degree than that of the other hydrogel groups.

### Antibacterial properties of multifunctional nanocomposite hydrogels *in vitro*

**Figure 4.**
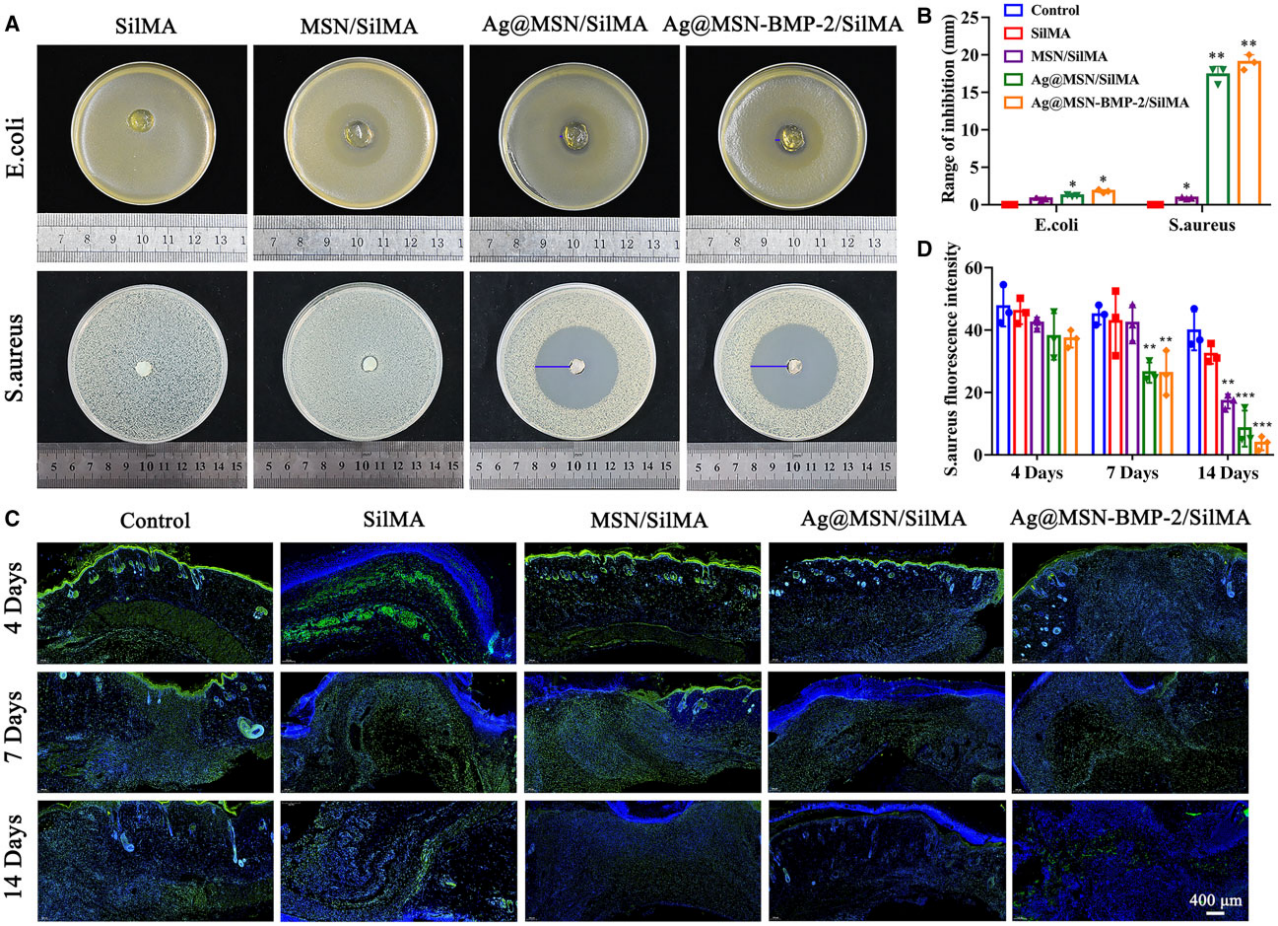
Antibacterial properties of Ag@MSN-BMP-2/SilMA hydrogels. (**A**) Digital images of surviving *E.coli* and *S.aureus* clones on agar plates after contact with SilMA, MSN/SilMA, Ag@MSN/SilMA and Ag@MSN-BMP-2/SilMA. (**B**) Inhibition range of hydrogel against *E.coli* and *S.aureus*. (**C**) Representative immunofluorescence images of the hydrogel scaffold at 4, 7 and 14 days after implantation. Anti-*S.aureus* expression and DAPI are shown in green and blue, respectively. (**D**) Statistical analysis of anti-*S.aureus* (*P <* *0.05, **P <* *0.01 and ***P <* *0.001, compared to the control group).

The growth of *E.coli* and *S.aureus* co-cultured with SilMA, MSN/SilMA, Ag@MSN/SilMA and Ag@MSN-BMP-2/SilMA was recorded to investigate the antibacterial properties of the multifunctional nanocomposite hydrogels. The inhibition rings on the two solid media represented the antibacterial properties of the hydrogels after 24 h. The largest inhibition rings corresponding to greater antibacterial properties was observed in the Ag@MSN/SilMA and Ag@MSN-BMP-2/SilMA groups (Fig. 4A). No inhibition rings were observed in the SilMA group. This suggested that SilMA cannot inhibit bacterial growth. The diameters of the inhibition ring in the Ag@MSN/SilMA group against *S.aureus* and *E.coli* were 17 and 1.5 mm, respectively (Fig. 4B) and the diameters of the inhibition ring in the Ag@MSN-BMP-2/SilMA group against *S.aureus* and *E.coli* were 19 and 2 mm, respectively (Fig. 4B). This showed that the inhibitory effect of the composite hydrogel was stronger against *S.aureus* than that of *E.coli*. Also, Ag NPs played an essential role in antibacterial properties.

Multifunctional nanocomposite hydrogels were used as surgical wound excipients to observe antimicrobial properties *in vivo*. Full-layer wounds were created on the dorsal side of SD rats and covered with SilMA, MSN/SilMA, Ag@MSN/SilMA and Ag@MSN-BMP-2/SilMA. Immunofluorescence detection of *S.aureus* was performed on traumatic tissues of rats at different time points. The hydrogels with Ag NPs showed less growth of *S.aureus* (green fluorescence) compared with hydrogels lacking Ag. This indicated that Ag contributed good antibacterial ability (Fig. 4C). The immunofluorescence intensity of *S.aureus* showed was significantly lower in Ag@MSN/SilMA and Ag@MSN-BMP-2/SilMA. This indicated that these hydrogels had a better antibacterial effect than that of the other groups (Fig. 4D).

### Immunogenicity and biodegradability of the multifunctional nanocomposite hydrogels *in vivo*

**Figure 5.**
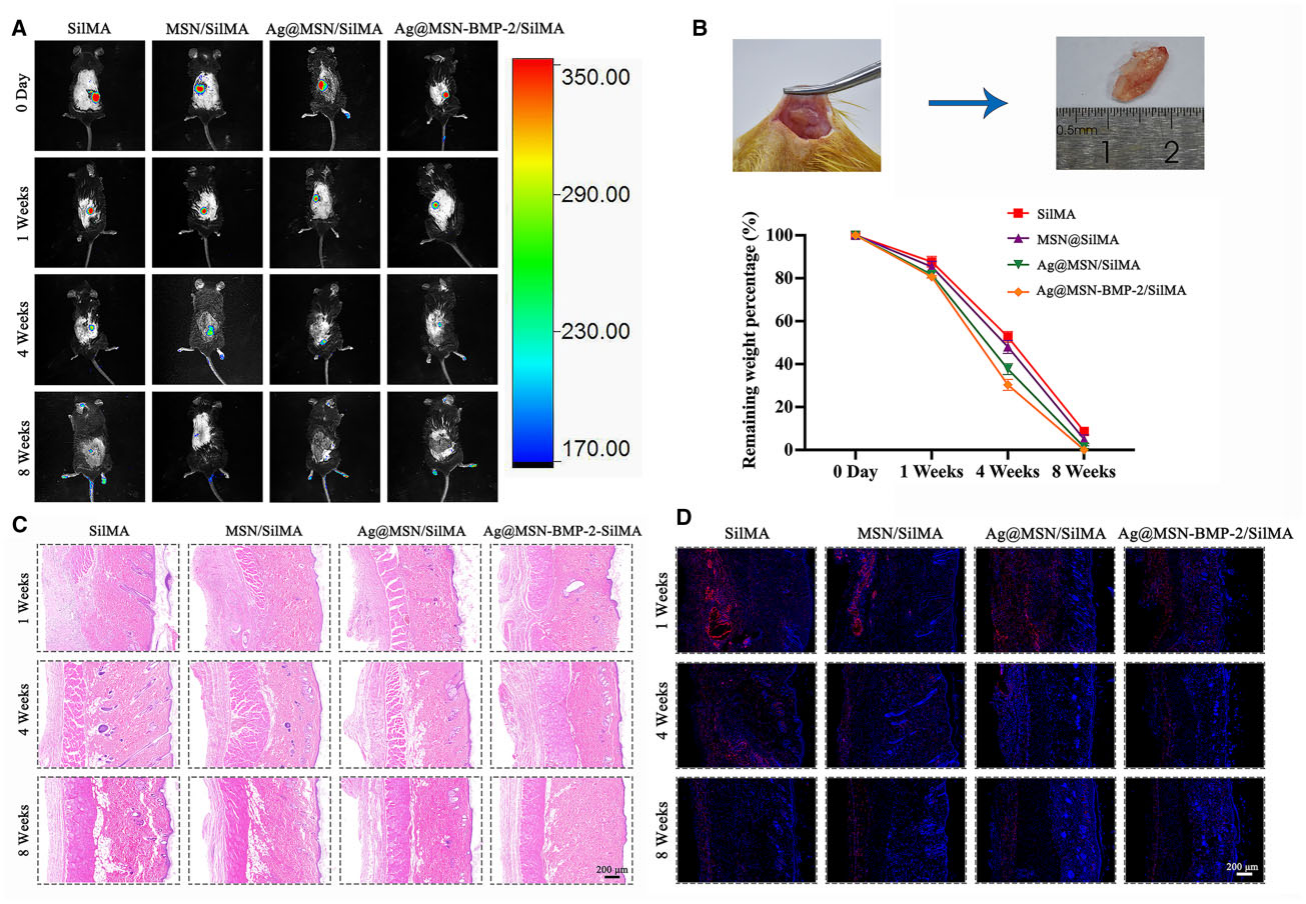
Hydrogel scaffolds implanted in animal subcutaneous tissue degrade *in vivo*. (**A**) Representative small animal live imaging images of hydrogel scaffolds degradation. (**B**) The *in vivo* degradation curve of each group was obtained by weighing the implant taken out between rat skin and subcutaneous fat at different times. (**C**) At various time points after implantation, H&E stained subcutaneous tissue around the hydrogel scaffold. (**D**) Representative immunofluorescence images of the skin tissue surrounding the hydrogel scaffold at various time points after implantation, with CD68 and DAPI expression shown in red and blue, respectively.

Fluorescently labeled SilMA, MSN/SilMA, Ag@MSN/SilMA and Ag@MSN-BMP-2/SilMA were implanted into the subcutaneous tissue on the back of C57BL/6 mice for small-animal live imaging to investigate the *in vivo* performance of the nanocomposite multifunctional hydrogels. The fluorescence intensity and positive area of the composite hydrogels were slightly lower than those of the SilMA group at week 1 (Fig. 5A). The SilMA group degraded more slowly than the other groups. The MSN/SilMA, Ag@MSN/SilMA and Ag@MSN-BMP-2/SilMA groups were completely degraded at week 8, with Ag@MSN-BMP-2/SilMA showing the fastest degradation rate. The residual implants were surgically removed for weighing, photographed to observe hydrogel degradation and the curves were plotted (Fig. 5B). Only the SilMA group showed incomplete degradation at week 8. These results suggested that the addition of NPs and bioactive factors improved the degradation of the composite hydrogels *in vivo*.

H&E staining and CD68 immunofluorescence staining were performed on the skin tissue sections around the composite hydrogels to assess their response to foreign materials (Fig. 5C and D). Initially, many inflammatory cells were found at the edges of the skin tissues due to the primary inflammatory response. The arrangement of myofilaments and collagen was disorganized, without any significant tissue edema or necrosis. The nanocomposite hydrogel scaffold degraded at weeks 4 and 8. This eliminated the inflammation and the skin histology returned to normal. Macrophages are important for wound healing, especially for relieving inflammation. CD68 belongs to the lysosomal glycoprotein family and is specifically expressed by tissue macrophages. CD68 expression (red fluorescence) was significantly lower in the Ag@MSN/SilMA and Ag@MSN-BMP-2/SilMA groups than that in the other groups. These findings revealed that the composite hydrogels had low immunogenicity.

### Synergistically enhanced bone regeneration *in vivo*

**Figure 6.**
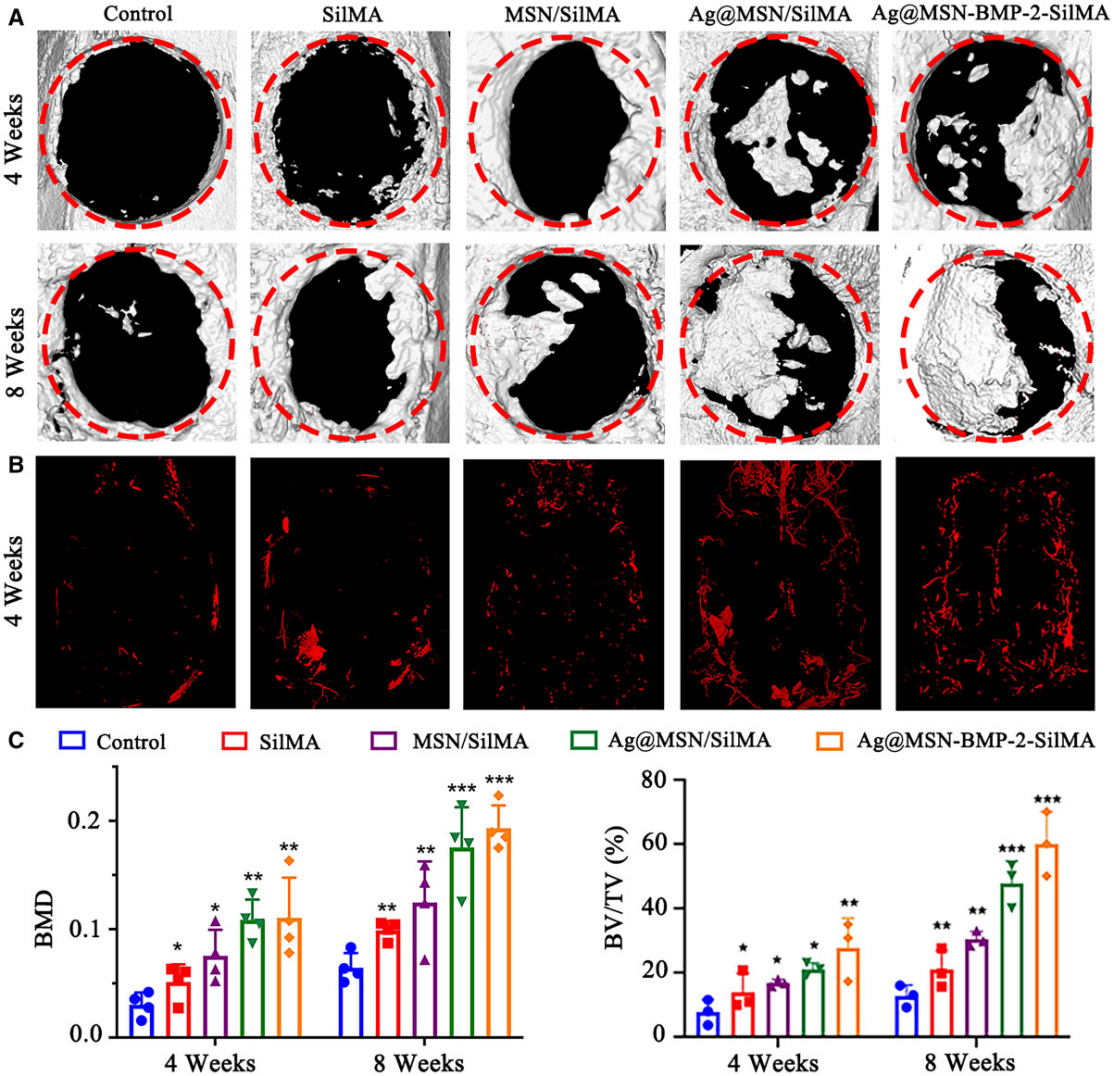
The ability of scaffolds to repair bone in the defect region at weeks 4 and 8 after implantation. (**A**) Representative 3D reconstructed images of the skull defect after micro-CT scanning. (**B**) Angiography infused with contrast media at 4 weeks postoperatively. (**C**) BMD and BV/TV in the defect region (*P <* *0.05, **P <* *0.01 and ***P <* *0.001, compared with the control group).

Bone formation of the nanocomposite multifunctional hydrogels was evaluated by micro-CT and H&E at weeks 4 and 8 after cranial surgery. In all groups, the 3D reconstructed micro-CT revealed that new bone was formed from the edge to the center (Fig. 6A). A 3D reconstructed micro-CT showed little new bone tissue in the control group at week 4, and a small amount of newly formed bone was visible around the nanocomposite hydrogels, especially in Ag@MSN-BMP-2/SilMA. The majority of defects in the control groups were empty at week 8 although a small amount of new bone was still present. This was indicative of self-healing bone tissues. New bone formation was significantly enhanced in the Ag@MSN-BMP-2/SilMA group compared with the other groups. This suggested that the NPs and BMP-2 provided a synergistic effect for osteogenesis. Bone mineral density in the Ag@MSN-BMP-2/SilMA group was 0.12 ± 0.13 and 0.21 ± 0.09 g/cm^3^ at weeks 4 and 8, respectively, while BMD values in the control group were 0.03 ± 0.05 and 0.07 ± 0.06 g/cm^3^ at week 4 and 8, respectively. Similarly, the BV/TV values in the Ag@MSN-BMP-2/SilMA group were 27.34 ± 4.21% and 58.7 ± 4.15% at weeks 4 and 8, respectively, while the BV/TV values in the control group were 7.88 ± 1.51% and 11.22 ± 2.47% at week 4 and 8, respectively (Fig. 6C). These results indicate that the osteogenic function of BMP-2 contributed to increased bone formation compared with the control group.

Angiography reconstructed by barium sulfate suspension perfusion showed a significant increase in vascularity around the implant at week 4. This indicated that the composite hydrogels induced angiogenesis in bone defects (Fig. 6B). Angiogenesis of the new bone was assessed by CD31 immunofluorescence staining, with blood vessels in defect areas showing red and round/oval structures (Fig. 9A). A small number of CD31-positive, small-diameter vascular vessels were observed in the control group at weeks 4 and 8, postoperatively. In contrast, higher proportions of these vessels were particularly observed in the composite hydrogel groups. Statistical analysis revealed that the number of new blood vessels in the Ag@MSN-BMP-2/SilMA group was much higher than that in the control group at weeks 4 and 8 (Fig. 9C).

**Figure 7.**
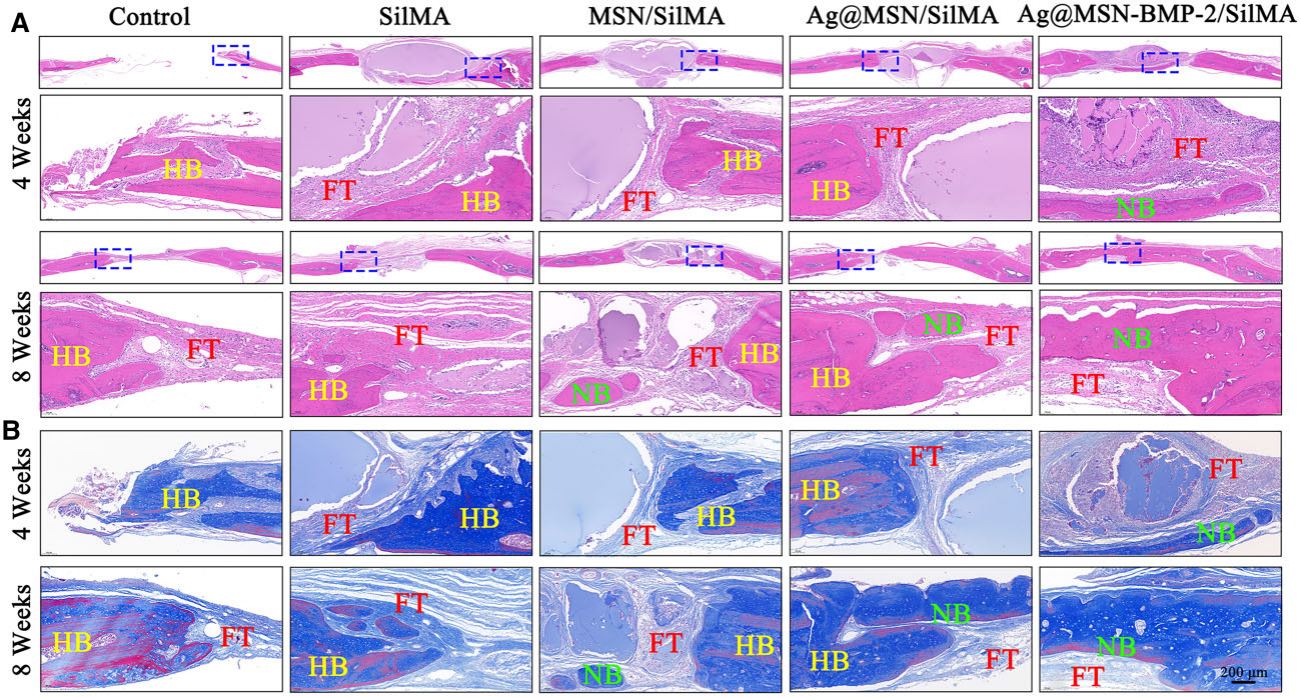
Examination of the bone defect region using histomorphology. (**A**) Representative images of the cranial defect stained with H&E and (**B**) Masson’s trichrome staining. HB, NB and FT stand for host bone, new bone and fibrous tissue, respectively.

To further assess the effectiveness of the composite hydrogels in promoting the repair of cranial defects, H&E staining and Masson’s trichrome staining were carried out 4 and 8 weeks after the scaffold was implanted. Little new bone tissue formed in the control group at week 4 according to H&E staining. The fibrous tissue arrangement was disturbed at week 8; however, soft tissue residue was formed, and a great deal of loose soft tissue developed around the defect. This caused discontinuity and hindered bone formation. Newly separated bone masses were observed in the Ag@MSN/SilMA group and the amount of new bone in the SilMA and MSN/SilMA groups was lower than that in the Ag@MSN-BMP-2/SilMA group. Specifically, a large amount of new bone matrix was observed in the Ag@MSN-BMP-2/SilMA group. This indicated that the defect was repaired and a larger volume of new bone was formed (Fig. 7A). Masson’s trichrome staining showed the same trend of new bone formation with the degradation of composite hydrogels and collagen deposition (Fig. 7B).

**Figure 8.**
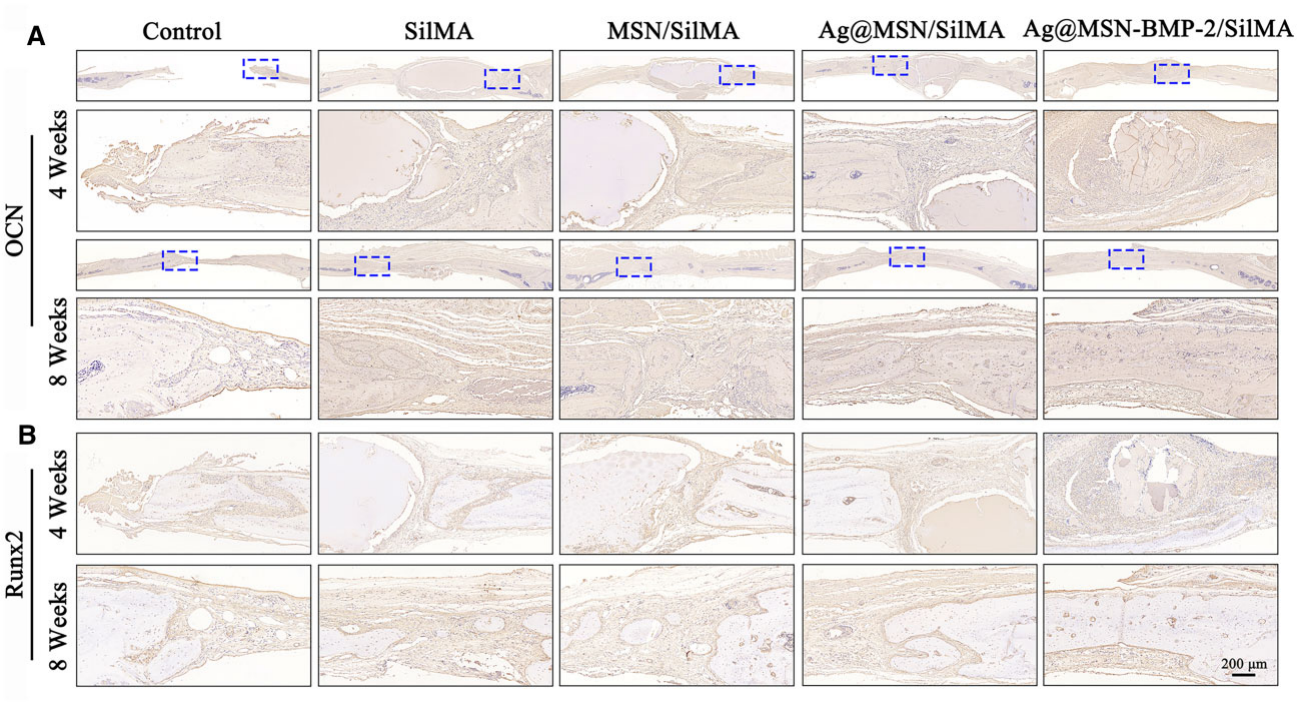
Bone repair immunohistochemical evaluation. (**A**) Representative immunohistochemical images of OCN and (**B**) RUNX2 expression in decalcified, defective bone in each group.

Immunohistochemistry and statistical analyses were used to confirm the expression of osteogenesis-related proteins during bone repair following scaffold transplantation (Fig. 8; Supplementary Fig. S9). Osteogenic-specific protein expression was significantly higher in Ag@MSN/SilMA, MSN/SilMA and Ag@MSN-BMP-2/SilMA (and especially in the Ag@MSN-BMP-2/SilMA group) in contrast to the control group at weeks 4 and 8. This correlated with the results of micro-CT and histological staining.

**Figure 9.**
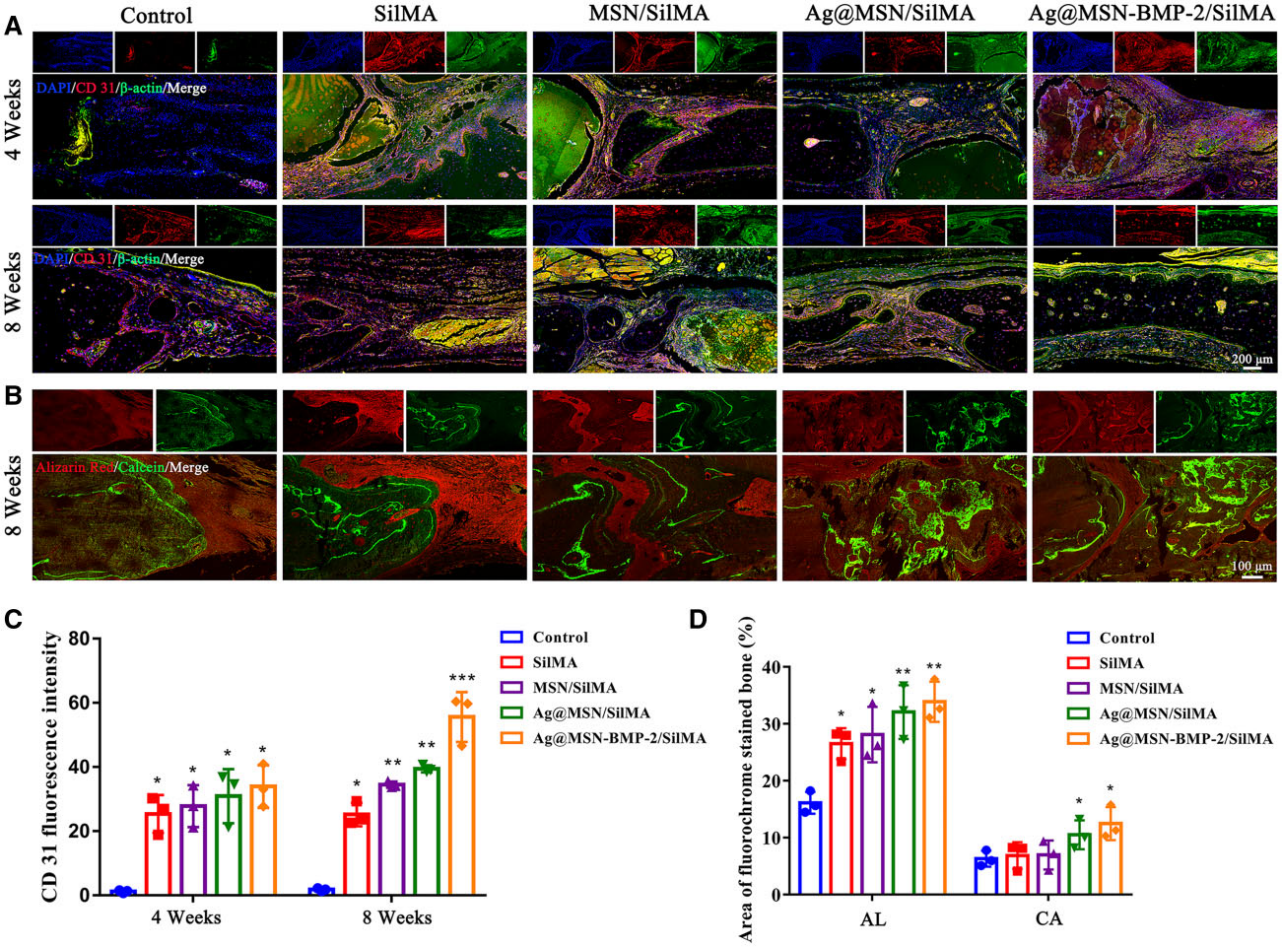
Histological analysis of the animal experiments. (**A**) Immunofluorescence staining of sections using the endothelial marker, CD31. (**B**) CLSM images of the calvarial defects’ resin sections (new bone with AL and CA injected after surgery was labeled red and green, respectively). (**C**) Quantitative analysis of CD31 immunofluorescence staining in different groups. (**D**) The fluorescence intensity indicates the rate of bone regeneration (*P <* *0.05, **P <* *0.01 and ***P <* *0.001, compared to the control group).

The fluorescence areas of AL (red) and CA (green) in the Ag@MSN-BMP-2/SilMA group were the largest among all groups, followed by the Ag@MSN/SilMA and MSN/SilMA groups according to CLSM plots (Fig. 9B) and semi-quantitative data (Fig. 9D) of fluorescent bands. These findings indicated that new bone was continuously being deposited at week 8 by *in situ* osteogenesis.

Therefore, the results of *in vitro* and *in vivo* osteogenesis, antibacterial activity and angiogenesis suggested that composite hydrogels promoted bone repair by inducing bone formation and vascularization.

## Discussion

Infection is a risk associated with the treatment of bone defects. Therefore, extensive research has been conducted on bone regeneration materials that can deliver local antibiotics [44]. However, only a few studies have looked into dual-use biomaterials that can do both of these things [35]. The great potential of novel inorganic–organic composite nanomaterials for bone regeneration has attracted attention [6, 45]. For example, silica NPs incorporated in hydrogel networks improve cell adhesion and stimulate osteogenesis in polymeric matrices [35, 46]. Furthermore, AgNPs have become attractive antibacterial agents for clinical application [47]. Controlling the release of drugs can reduce the toxic side effects of drugs to some extent [48]. This study prepared MSN encapsulating Ag NPs (Ag@MSN) with uniform spherical size and mesoporous structure, loaded it with BMP-2, and encapsulated it using light-cured silk protein hydrogels for optimal slow-release properties. The tissue remodeling process was systematically explored through the promoting effects of the composite hydrogels on BMSC vascularization, the inducibility of BMP-2 in bone stimulation of BMSCs, and the antibacterial capacity of the Ag^+^-loaded multifunctional complex.

The prepared nanocomposites had good porosity, satisfactory mechanical properties and good cell adhesion. The nanocomposite hydrogels all showed similar sponge-like structures with a porous morphology (Fig. 2B). This indicated that the addition of MSN, Ag@MSN and Ag@MSN-BMP-2 did not change the porous structure of the SilMA hydrogel. In addition, no agglomeration of NPs was observed in the high-magnification images, whereas energy spectrum analysis indicated that the nanomaterials were well dispersed. Moreover, the addition of nanomaterials increased the number of pores and decreased the pore size, while the interconnecting porosity structures aided cell and blood vessel development as well as the flow of nutrients and metabolic wastes. Swelling is a fundamental property of hydrogels, and hydrogels with good swelling capabilities promote the exchange of nutrients and metabolic wastes within cells [6]. The water absorption of the SilMA hydrogels exceeded 1000 wt% after swelling in PBS for 10 h. In addition, the nanocomposites have a suitable pore size and enhanced mechanical stability to ensure a balanced cell survival microenvironment and humidity for cell growth [6]. These outcomes showed that the Ag@MSN-BMP-2/SilMA composite hydrogels had high water content and a porous structure that contributed to the growth of stem cells. The 3D microenvironment is essential for regulating the direction of MSC differentiation [49]. Bone marrow stem cells in the microenvironment tended to differentiate toward adipogenesis and osteogenesis at 2–5 and 10–30 kPa, respectively. The elastic modulus of the composite hydrogel was approximately 15 kPa. This is suitable for MSC growth and induction of MSC differentiation toward osteogenesis [5, 50].

Almost all BMSCs survived on the nanocomposite materials after 1, 4 and 7 days of culture. This verified the biocompatibility of the hydrogels (Fig. 3A). The slight decrease in the survival rate of each group observed on the fourth day might be related to cell metabolism. The ALP levels in the nanocomposite materials indicated that early osteogenic activity occurred. Calcium nodules formed by osteoblasts are also markers of osteoblasts. Since the chromogenic reaction between alizarin red and calcium can form a deep red compound, the calcium nodules deposited outside the osteoinductive cells were also stained deep red. The strongest osteogenic differentiation was observed in the Ag@MSN-BMP-2/SilMA group after 7 and 14 days (Fig. 3C). These results suggested that BMP-2 grafted monodisperse silica promoted mineralized matrix formation and further improved the mineralization efficiency of the composite hydrogels.

Ag@MSN/SilMA and Ag@MSN-BMP-2/SilMA showed significant inhibition of *S.aureus* and *E.coli*, whereas scaffolds without Ag^+^ had insignificant inhibition based on an inhibition ring test (Fig. 4A). In addition, the inhibition of *S.aureus* by the composite scaffolds was superior to that of *E.coli*. Ag’s inhibitory impact is primarily dependent on the release of Ag^+^ which penetrates the bacteria to interrupt the function of the cell membrane and DNA replication; it also catalyzes the formation of reactive oxygen species which leads to cell death [47]. Based on the Ag^+^ release profile, the composite scaffold had a sustained Ag^+^ release capacity and inhibited Gram-positive and Gram-negative bacteria (Supplementary Fig. S6). The uniform distribution of Ag NPs and controlled release of Ag^+^ in this study are prerequisites of functional Ag-containing materials in tissue repair [49, 51]. The *in vivo* antibacterial fluorescence assay of trauma sections in rats showed the same antibacterial trend as that of the *in vitro* inhibition ring test. This further validated the potential of composite hydrogels for effective antibacterial therapy (Fig. 4C). These results indicated that the inherent antimicrobial ability of the Ag@MSN-BMP-2/SilMA hydrogel accelerated the pathogen-killing process. In addition, the hydrogel at the wound sites may help promote the healing of infected bone defects through its hemostatic function, adsorption of exudate, function as a barrier for microorganisms, gas exchange and cellular scaffolding [2, 52, 53].

A rat skull defect model was established to further verify the ability of nanocomposite hydrogels to induce *in situ* bone regeneration *in vivo* and to assess their angiogenic and osteogenic abilities. Rats in the Ag@MSN-BMP-2/SilMA group had the largest area of new bone formation according to micro-CT results (Fig. 6A). This was confirmed by the quantitative analysis of BMD and BV/TV (Fig. 6C). Ag@MSN-BMP-2/SilMA had the highest capacity for new bone generation, with increased bone trabecular structures, a significant amount of new bone generated in the defect location, and highly mature collagen in the bone tissues according to the H&E and Masson’s staining results. In contrast, less bone formation was detected in the postoperative control group at weeks 4 and 8. This may have resulted from the absence of scaffolding in the defects to support osteoblast adhesion and migration. Meanwhile, the residual hydrogel was observed around the newly formed bone tissue with no significant inflammation or immunological rejection at week 4. The hydrogels were completely absorbed in the Ag@MSN/SilMA and Ag@MSN-BMP-2/SilMA groups at week 8. This correlates with the results shown in Fig. 5A. In addition, the composite hydrogels exhibited a slow release of BMP-2 that facilitated the long-term regulation of bone regeneration (Supplementary Fig. S7). Immunohistochemical staining revealed a similar pattern (Fig. 8) and the CLSM images of AL and CA-labeled fluorescent bands (Fig. 9B and D); sustained deposition of newly formed bone was found at week 8 together with the strongest fluorescence expression in the composite hydrogels containing BMP-2.

Angiogenesis and osteogenesis are considered equally important for bone regeneration strategies in tissue engineering since bone is a highly vascularized and mineralized tissue [54]. There was a significant increase in vascularity around and within the bone defects reconstructed by perfusing the barium sulfate suspension at week 4 (Fig. 6B). Angiogenesis of the new bone was observed at weeks 4 and 8 as many new vascular lumens within or around the new bone were accompanied by the appearance of CD31-stained endothelial cells (Fig. 9A). Si ions can stimulate osteogenesis and angiogenesis by regulating stem cell migration and differentiation [55]. These results suggest that BMP-2 peptide-bound MSNs synergistically induced *in situ* bone formation *in vivo* and the presence of Ag enhanced this ability. Covalent immobilization of BMP-2 on Ag@MSNs and encapsulation by SilMA preserved the biological activity of BMP-2 by protecting the peptide cleaved by proteases *in vivo*. Furthermore, the adverse side effects caused by the sudden release of BMP-2 at high doses were significantly reduced. BMP-2 induction results in the porous hydrogel structure promoting nutrient supply and tissue growth for rapid bone regeneration. BMP-2’s gradual release gifted the composite hydrogels with sustained bioactivity and established a bioactive milieu that allowed stem cells to adhere, multiply and differentiate into osteogenic tissue. However, bone formation is sequentially and synergistically controlled by many growth factors; therefore, other bioactive factors are often added as synergistic factors to further enhance osteogenic differentiation. In this study, Ag was loaded into the mesopores of BMP-2 peptide-grafted MSN and continuously released from the pores to promote osteoblast differentiation. These results suggest that Ag@MSN-BMP-2/SilMA composite hydrogels induce angiogenesis during bone defect repair and provide more nutrition for subsequent bone formation. For tissue engineering, it is particularly necessary to design biomaterials with mechanical properties and degradation rates that match the new tissue. Most importantly, virtually current research on filamentous protein hydrogels has focused on cellular and small animal models, while less research has been done on large animal models. Therefore, more research is needed to obtain clinical trial approval and accelerate clinical implementation for future treatment of bone injury repair.

## Conclusion

To create an injectable nanocomposite multifunctional hydrogel system, Ag was injected into the mesopores of BMP-2 peptide-grafted MSN and combined with SilMA hydrogel. BMP-2 was firmly immobilized in the hydrogel by covalent binding with Ag@MSN, which was characterized by the sustained release of the peptide that acted as a synergistic factor with MSN to maintain the biological activity of BMP-2 and significantly prolonged drug release. The material showed good biocompatibility *in vitro*, enhanced BMSCs differentiation into osteogenic cells and aided in bone regrowth. This composite scaffold had considerable degradability and low immunogenicity *in vivo*, making it more suitable for bone tissue engineering. It achieved efficient bactericidal ability against *E.coli* and *S.aureus in vitro*, with superior antibacterial activity *in vivo*. The Ag@MSN-BMP-2/SilMA hydrogel possessed good *in situ* bone regeneration ability in a rat skull defect model. In conclusion, the constructed nanocomposite hydrogel technology demonstrated tremendous promise for promoting *in situ* bone tissue regeneration.

## Supplementary Material

rbad046_Supplementary_Data
