# Supplementary material for: A multifunctional nanocomposite hydrogel with controllable release behavior enhances bone regeneration
Source: Regen Biomater. 2023 Apr 28;10:rbad046. doi: 10.1093/rb/rbad046 (PMC10243836; doi:10.1093/rb/rbad046)
Supplement: rbad046_Supplementary_Data [file rbad046_supplementary_data.docx]

**Supplementary data**

A multifunctional nanocomposite hydrogel with controllable release behavior enhances bone regeneration

Yingji Mao^1,2,†^, Yiwen Zhang^1,3,†,*^, Ying Wang^1^, Tao Zhou^1^, Bingxu Ma^1^, Pinghui Zhou^1,2,*^

^1^ Department of Orthopedics and Department of Plastic Surgery, The First Affiliated Hospital of Bengbu Medical College, Bengbu, Anhui 233004, China

^2^ Anhui Province Key Laboratory of Tissue Transplantation, and School of Life Sciences, Bengbu Medical College, Bengbu, Anhui 233030, China

^3^ Department of Plastic Surgery and Burn Center, Second Affiliated Hospital, Plastic Surgery Institute of Shantou University Medical College, Shantou, Guangdong 515063, China

**^†^** Yingji Mao and Yiwen Zhang contributed equally to this work.

***Corresponding Authors:**

Yiwen Zhang, Department of Plastic Surgery, The First Affiliated Hospital of Bengbu Medical College, Bengbu, Anhui 233004, China; Tel: +86-552-3175396; Email: zhangyiwen0305@163.com;

Pinghui Zhou, Department of Orthopedics, The First Affiliated Hospital of Bengbu Medical College, Bengbu, Anhui 233004, China; Tel: +86-552-3086021; Email: [zphdoctor@126.com](mailto:zphdoctor@126.com).

## 1 Material and methods

### The preparation of composite hydrogels

#### 1.1.1 The preparation of MSN

The MSN was prepared with a modified method according to a previously reported study [1]. 1.0 g CTAB and 0.28 g NaOH were dissolved in 480 mL of deionized water, and the mixed solution was heated to 80°C with magnetic stirring to accelerate dissolution. Then, 5.0 mL TEOS was added dropwise to the surfactant solution under stirred vigorously at 80°C for 2 h. The mixed solution was transferred from the flask to a centrifuge tube and was then centrifuged at 15,000 rpm to obtain mesoporous silicon containing the templating agent CTAB. The mesoporous silicon was washed with ethanol and centrifuged 3-5 times and dried under vacuum for 12 h at 60°C to obtain MSN with the template removed.

#### 1.1.2 The preparation of Ag@MSN

The MSN was prepared as described above. After preheating the mixed solution of CTAB and NaOH at 80°C for 20 min, formaldehyde (1 M, 0.3 mL) and AgNO_3_ (0.1 M, 1 mL) were added with constant stirring. TEOS (0.535 mL) and ethyl acetate (2 mL) were added dropwise to the reaction solution. All reagents were stirred at 80°C for 2 h. The products were collected by centrifugation and washed with ethanol. Finally, the template was removed by an efficient ion exchange method, and the purified nanoparticles were dispersed in an ethanol solution (60 mL) containing NH_4_NO_3_ (60 mg) in an ultrasonic bath for 2 h. The above steps were repeated three times to obtain the complete removal of the template Ag@MSN [2, 3].

**1.2 Characterization of MSN and Ag@MSN**

**1.2.1 Transmission electron microscope (TEM)**

The morphologies of monodisperse silica nanoparticles, Ag^+^-loaded aminated mesoporous silica, and nano silver were observed by transmission electron microscopy (TEM, JEM-2100 microscope) and analyzed.

#### 1.2.2 Fourier transform infrared spectroscopy (FTIR)

FTIR spectra were obtained using an FTIR spectrophotometer (Nicolet 6700, USA) to identify the different functional groups of MSN and Ag@MSN, with a range of 400 to 4000 cm^-1^.

#### 1.3 The preparation of FITC-labeled BMP-2

To obtain FITC-labeled BMP-2, FITC-APTES was synthesized by reacting FITC with 3-aminopropyltriethoxysilane (APTES) in 1.0 mL of ethanol protected from light for 24 h. 0.5 mL of FITC-APTES solution was added before TEOS during the synthesis of BMP-2. Then, the surfactant-free FITC-labeled BMP-2 was dried under a vacuum and photographed under a fluorescence microscope to validate the successful grafting.

#### 1.4 Cell morphological observation

For cell morphological observation of Ag@MSN-BMP-2, BMSCs were seeded on the surface at a density of 5×10^4^ cells in 6-well plates. After 48 and 72 h of culturing with 100 ng/mL Ag@MSN-BMP-2-FITC, cells were fixed by 4% paraformaldehyde (PFA) and were permeabilized with 0.5% TritonX-100 (Sigma-Aldrich, USA) for 5 min. Then cells were stained with Rhodamine (Yeasen, China) and 4,6-diamidino-2-phenyindole dilactate (DAPI, Life Technologies, USA) for 5 min to label the cytoskeleton and nucleus, respectively. The cytoskeletal arrangements were visualized under a laser confocal microscope (Olympus Corporation, Japan).

## Reference

1. Zhou X, Feng W, Qiu K, Chen L, Wang W, Nie W, Mo X, and He C. BMP-2 Derived Peptide and Dexamethasone Incorporated Mesoporous Silica Nanoparticles for Enhanced Osteogenic Differentiation of Bone Mesenchymal Stem Cells*.* *ACS Appl Mater Interfaces* **2015**;7:15777-89.

2. Han L, Wei H, Tu B, and Zhao D. A facile one-pot synthesis of uniform core-shell silver nanoparticle@mesoporous silica nanospheres*.* *Chem Commun (Camb)* **2011**;47:8536-8.

3. Wang, Y, Ding X, Chen Y, Guo M, Zhang Y, Guo X, and Gu H. Antibiotic-loaded, silver core-embedded mesoporous silica nanovehicles as a synergistic antibacterial agent for the treatment of drug-resistant infections*.* *Biomaterials* **2016**;101:207-16.

**Figures S1-S9**


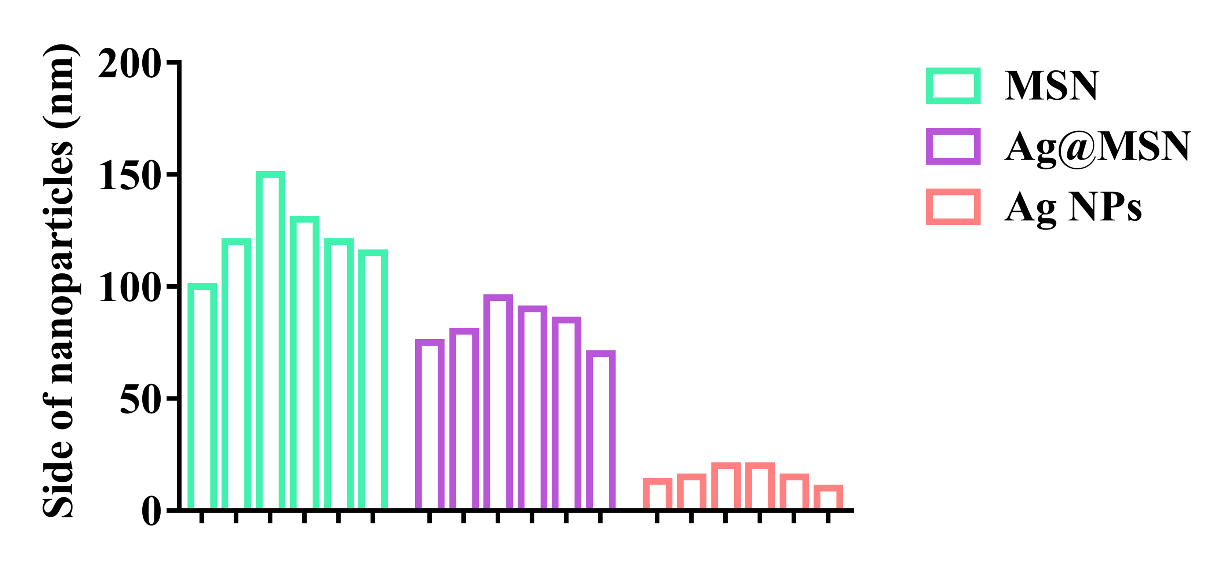


**Figure S1** Size distribution of MSN, Ag@MSNs, and Ag NPs measured from TEM images.


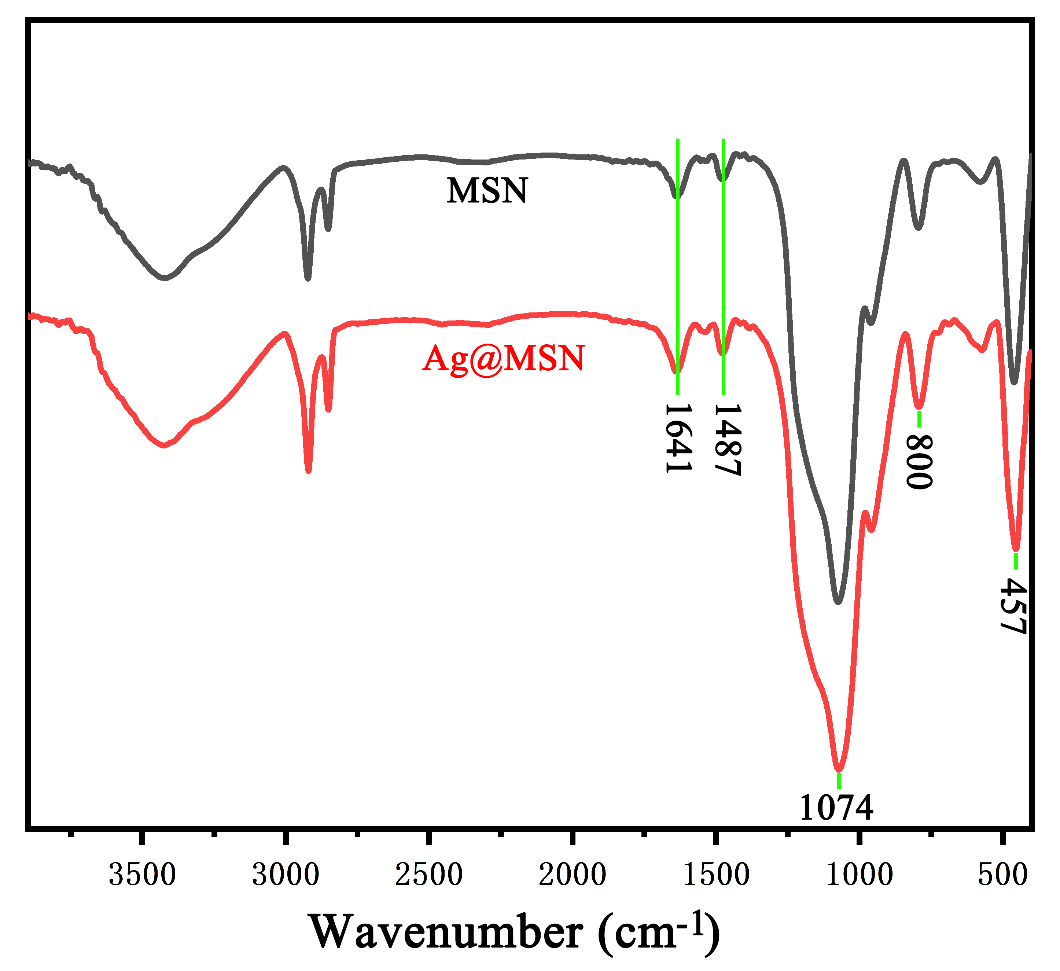


**Figure S2**. FT-IR spectra of MSN and Ag@MSNs.


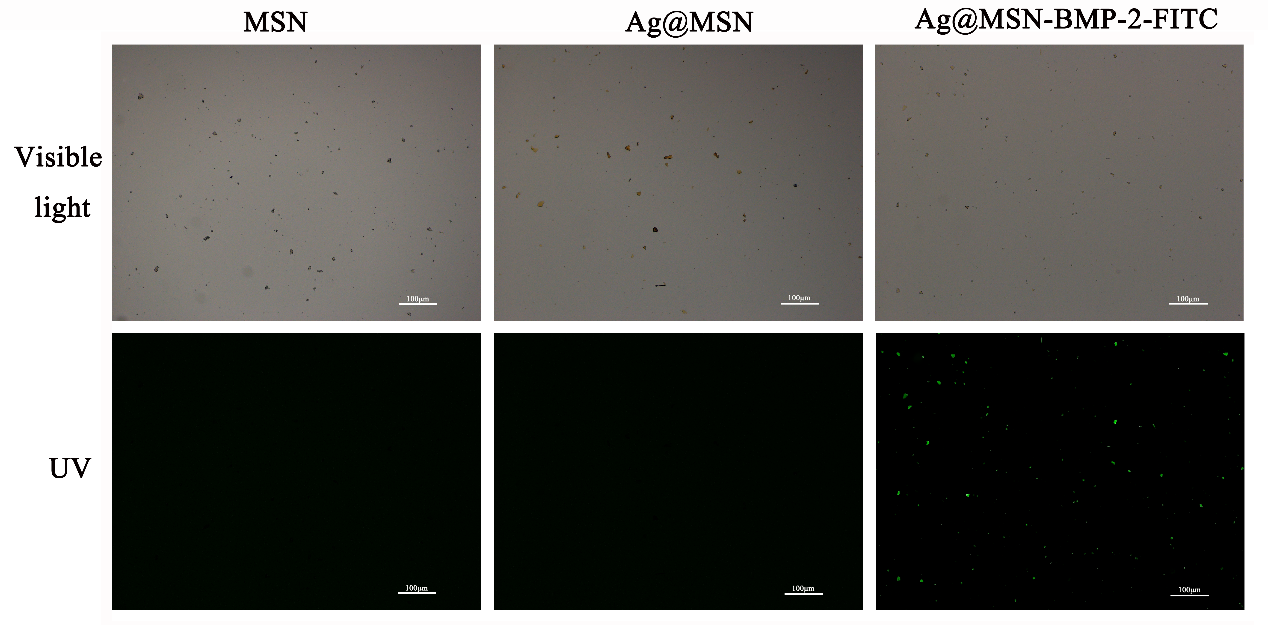


**Figure S3.** Bright-field and fluorescence microscopy images of MSN, Ag@MSN, and Ag@MSN-BMP-2-FITC, respectively.


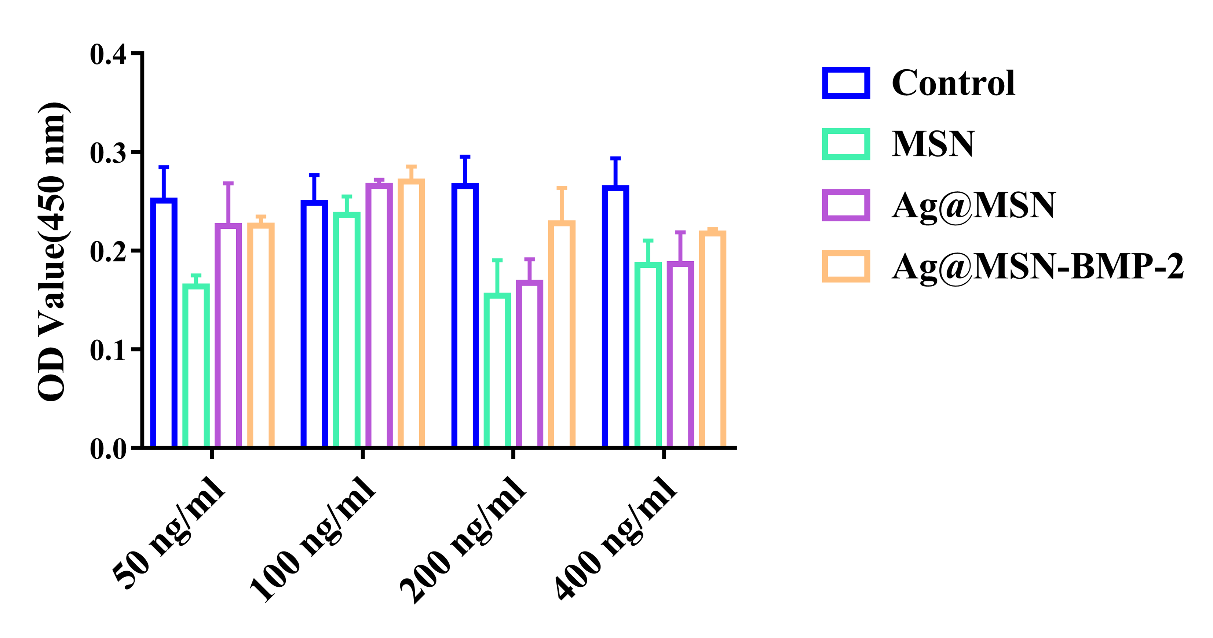


**Figure S4** Proliferation of BMSCs cultured on different concentration nanoparticles after 3 days.


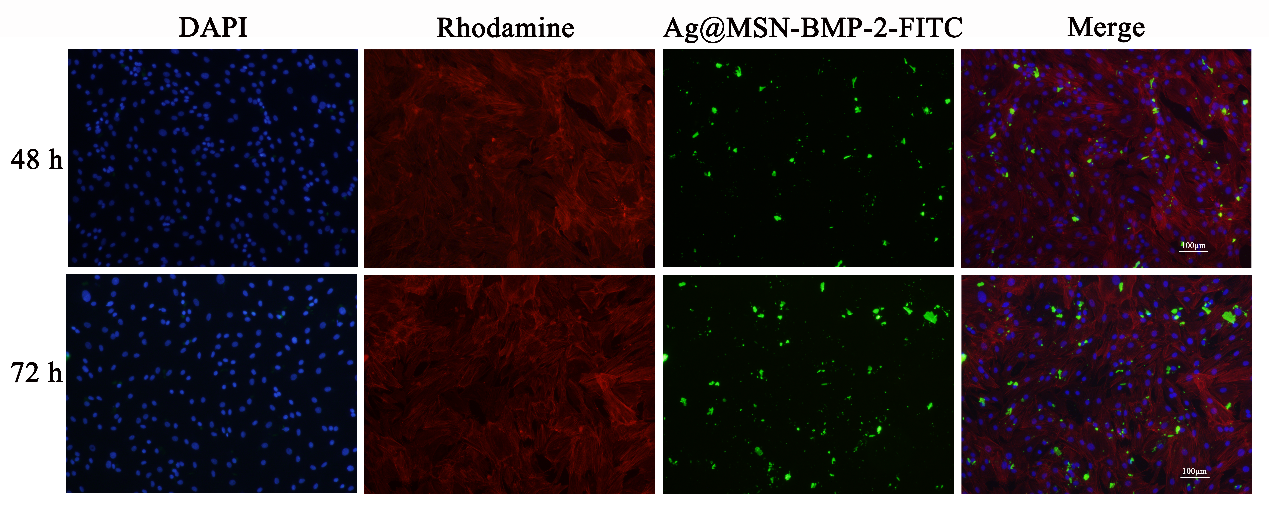


**Figure S5** CLSM images of BMSCs incubated with the same amount of Ag@MSN-BMP-2-FITC (green) for 48 and 72 h. Actin filament in the cell cytoskeleton was stained with phalloidin (red), and nuclei were stained with DAPI (blue).


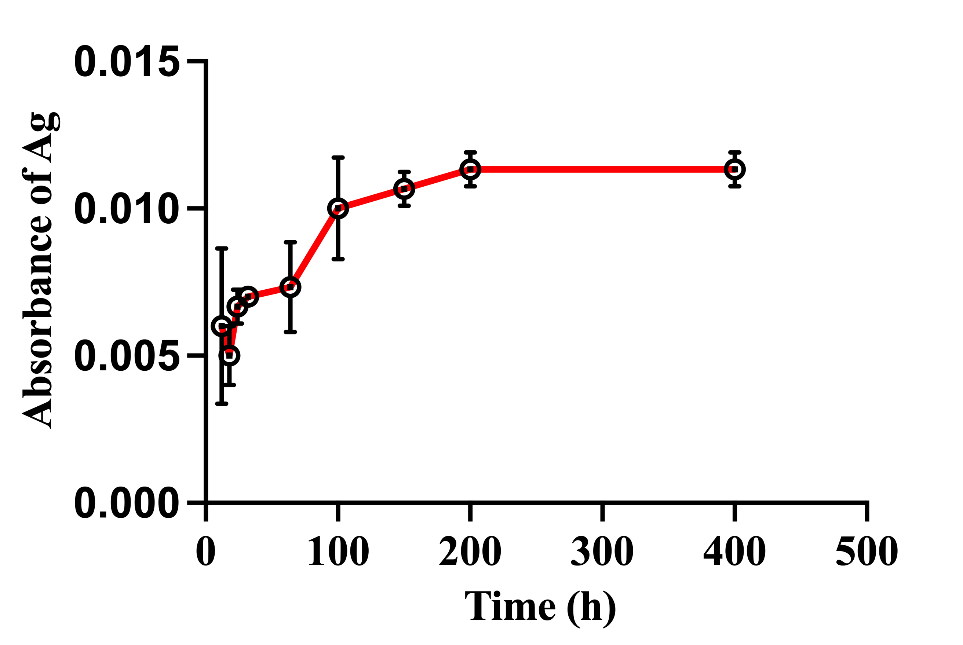


**Figure S6** The consumption profiles of Ag content from Ag@MSN-BMP-2-FITC/SilMA in LB medium at 37 °C.


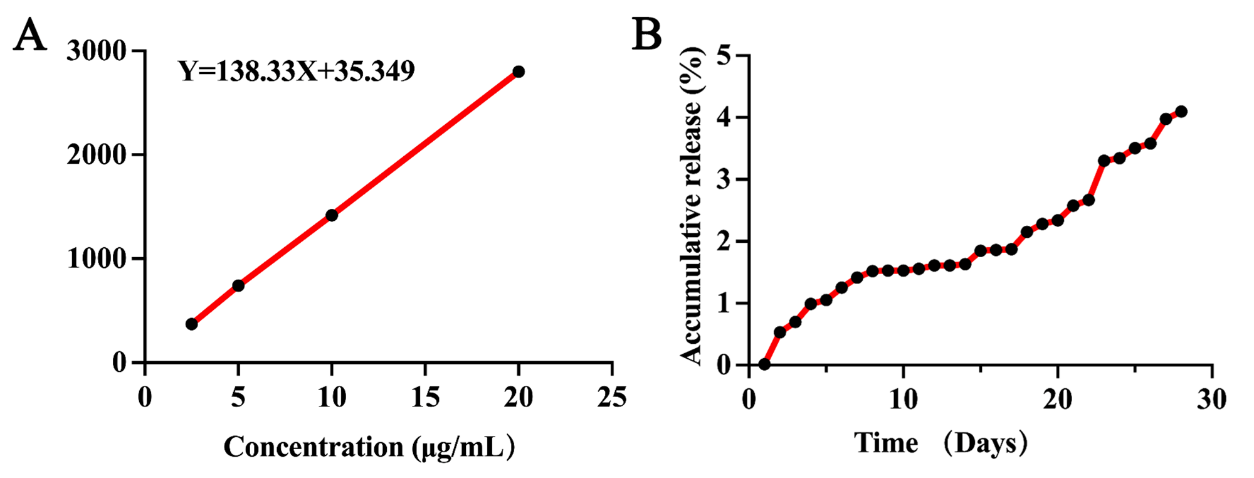
**Figure S7** (A) Standard curve of BMP-2-FITC peptide (FITC-KIPKASSVPTELSAISTLYL sequence) aqueous solution. (B) BMP-2 release spectrum of Ag@MSN-BMP-2-FITC/SilMA at 37°C.


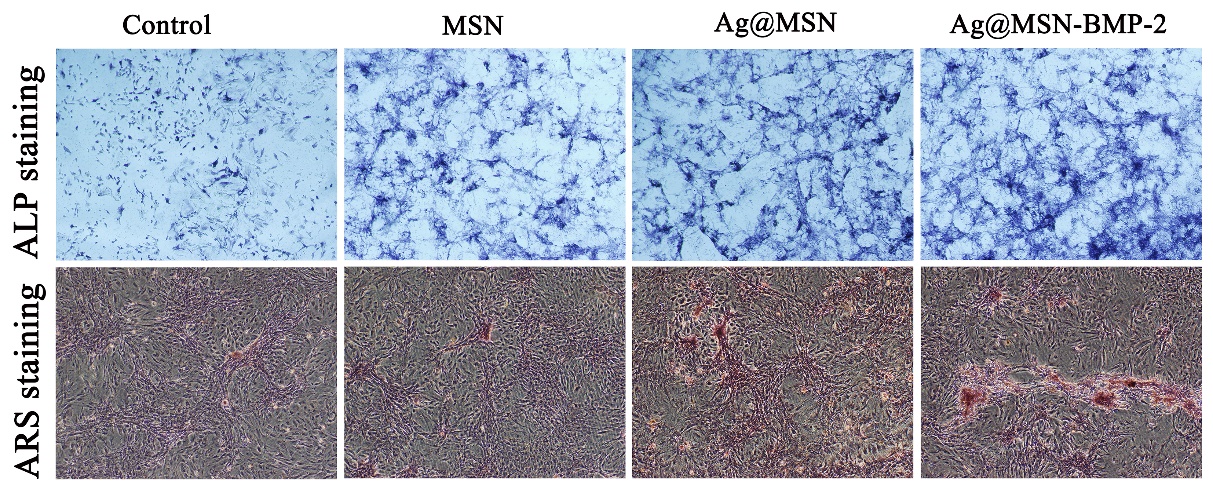


**Figure S8** The ALP and ARS staining images of BMSCs cultured with nanoparticles after induction for 7 days and 14 days.


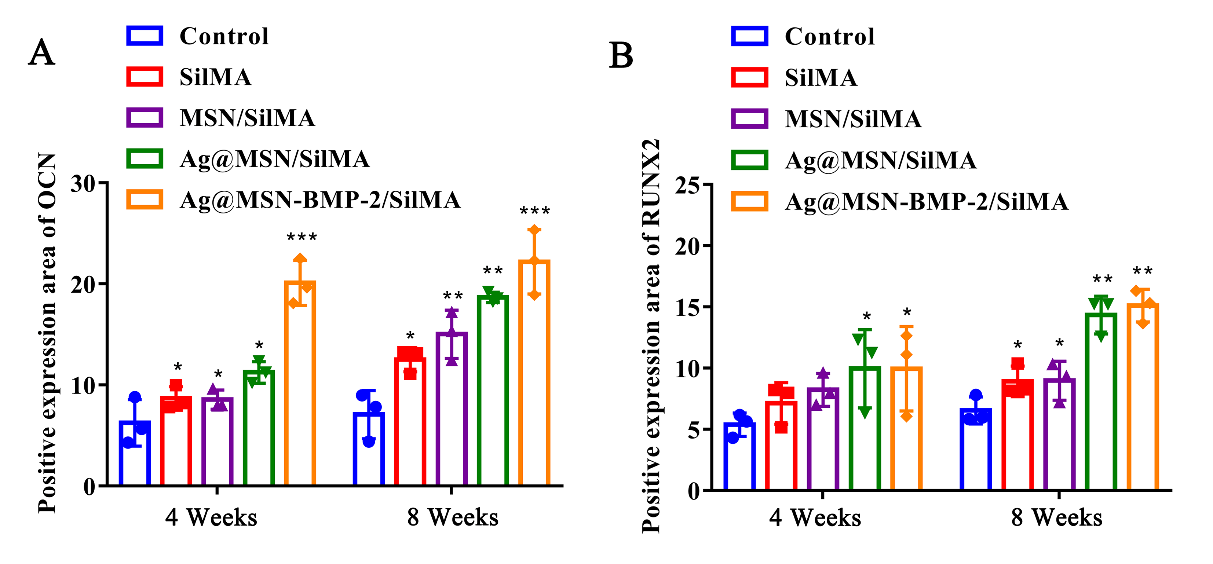


**Figure S9** (A and B) Quantitative analysis of the expression of OCN and RUNX2. (* *p* < 0.05, ** *p* < 0.01, and *** *p* < 0.001, compared to the control group)
